# Gut microbiome plasticity explains the altitudinal distribution pattern and adaptability in a small mammal species (*Apodemus draco*)

**DOI:** 10.1128/spectrum.02388-25

**Published:** 2025-11-20

**Authors:** Yang Yun, Chao Duan, Xingcheng He, Ruixiang Tang, Yue Lan, Muyang Lu, Tianjiao Liu, Xing Fan, Zhenxin Fan, Jianghong Ran

**Affiliations:** 1 Key Laboratory of Bio-Resources and Eco-Environment of Ministry of Education, College of Life Sciences, Sichuan University 12530, Chengdu, China; 2 Sichuan Key Laboratory of Conservation Biology on Endangered Wildlife, College of Life Sciences, Sichuan University 12530, Chengdu, China; 3 Chengdu Management Branch of Giant Panda National Park, Chengdu, China; Chengdu University, Chengdu, Sichuan, China

**Keywords:** small mammal, gut microbiome, altitudinal distribution pattern, vegetation cover, high-altitude adaptation

## Abstract

Altitudinal distribution patterns of species, a central focus of ecology, predominantly focus on environmental factors and only rarely on the host’s intrinsic adaptive capacity. Particularly, the role of gut microbiota has not yet been studied. Here, we used the wild South China Field Mouse (*Apodemus draco*), a widely distributed small mammal species, as the study subject to investigate the altitudinal distribution pattern of the species and assess how gut microbiota contributes to the formation of this pattern. A total of 219 wild samples were captured in the middle section of the Qionglai Mountains, China, and 121 adult individuals were selected for metagenomic sequencing (e.g., gut microbial diversity, network topology, composition, and functional profiles). Vegetation cover of each sampling quadrat was assessed using Normalized Difference Vegetation Index. Our results indicate that *A. draco* exhibited a hump-shaped altitudinal distribution, but the peak abundance of *A. draco* corresponds to lower vegetation cover of habitats. Gut microbial diversity, complexity, robustness, energy harvesting ability, and carbohydrate utilization capacity all peaked at the mid-altitude zone, matching the host’s spatial distribution pattern. Furthermore, the gut microbiome in high-altitude *A. draco* populations facilitates host acclimatization in extreme high-altitude niches by enhancing energy harvesting, hypoxia tolerance, and pathogen resistance.

**IMPORTANCE:**

We propose for the first time that the gut microbiome serves as a pivotal factor in structuring the altitudinal distribution pattern of species and further reveal a gut microbiota-mediated adaptive strategy underlying mammalian high-altitude adaptation. These results demonstrate that the gut microbiome fundamentally facilitates host adaptation to ecological niches. The study provides a novel insight into the factors of species’ spatial distribution from a gut microbiota perspective.

## INTRODUCTION

Understanding the distribution patterns of animal species along environmental gradients and their underlying mechanisms has long been a key topic in ecological research. Altitude, as a multifactorial environmental gradient, structures species distribution patterns across spatial scales through complex environmental filtering and intrinsic physiological mechanisms of species, including climate, space, biotic interactions, and adaptive evolution (1). Small mammals constitute an important component of global vertebrate biomass and play fundamental roles in key global ecological processes (2). Currently, numerous studies to date endorse the concept that the altitudinal distribution pattern of non-volant small mammals in species richness and diversity conforms to the pattern of mid-altitudinal peak (1, 3, 4). However, while previous studies on the mechanisms of altitudinal distribution patterns of small mammals have traditionally emphasized environmental factors, much less is known about the factors of species’ intrinsic adaptive capacity, especially the role of gut microbiota in structuring the altitudinal distribution patterns of small mammals has not yet been studied.

As the host’s second genome, accumulating evidence demonstrates that gut microbiota critically modulates host health by maintaining physiological homeostasis and influencing disease susceptibility (5, 6). Evidence from wild mammals, birds, reptiles, and amphibians indicates that gut microbiota fundamentally facilitates host adaptation to ecological niches (e.g., environments and diets) through long-term coevolution (7–16). In addition, convergent features were identified in the gut microbiota of high-altitude animals, including adaptations to hypoxia, low temperature, and limited resources (13, 14, 17–20). Notably, compared to host genomes, gut microbiota can respond to environmental changes more rapidly and flexibly, thereby facilitating host adaptation to environmental fluctuations (21, 22). Therefore, gut microbiota plays an indispensable role in the environmental adaptation of species.

The distribution patterns of species along environmental gradients are the result of adaptations or dependencies on particular environments during long-term evolution. Adaptability, especially in physiological or morphological traits, is also a key driver of distribution patterns of species (23). However, current research on the altitudinal distribution of small mammals mainly focused on species richness and diversity, with mechanistic explanations often restricted to environmental factors, neglecting species’ intrinsic adaptability (1, 4). Thus, we argue that investigating species’ altitudinal distribution patterns from the perspective of adaptability at the species level is essential for a comprehensive understanding of distribution patterns.

South China Field Mouse (*Apodemus draco*), an endemic rodent species in China, is widely distributed from west to east (24). The species is primarily found in forests, thickets, and meadows and has been observed to inhabit a wide range of altitudes, ranging from 800 m to above 3,000 m (25). Importantly, widespread species demonstrate greater ecological adaptive capacity than narrowly distributed species, exhibiting remarkable environmental tolerance across environmental gradients through enhanced phenotypic plasticity. In particular, gut microbiota plasticity has been shown to be critical for maintaining host fitness and survival (11, 19, 22, 26). Therefore, *A. draco* provides an ideal subject to investigate how gut microbiota determines species’ altitudinal distribution by enhancing or modulating their ecological adaptive capacity.

Here, we report the altitudinal distribution of *A. draco* and, for the first time, propose a potential role of the gut microbiome in structuring species’ spatial distribution. Meanwhile, our findings further advance the understanding of the contributory role of the gut microbiome in wild mammals adapted to high-altitude environments. Overall, the study provides a novel insight into the factors influencing species’ spatial distribution from a gut microbiota perspective.

## MATERIALS AND METHODS

### Sample collection

In total, 219 *A*. *draco* samples were captured using snap traps in 2022 and 2023 (May–August) in the middle section of Qionglai Mountains, China, which covers Jiguan Mountain and Xiling Snow Mountain. Quadrats were set up along altitude from low to high according to the topography to cover different vegetation and habitat types as much as possible, and the straight-line distance between adjacent quadrats is not less than 100 m. A total of 64 quadrats in the study were set up along an altitude ranging from 1,400 to 4,400 m. Each quadrat size was set at 20 × 20 m with 50 snap traps, and fresh peanut kernels were used as bait. Snap traps were placed in each quadrat for 3 days, and trapped samples were collected every morning. The study area and quadrats are shown in the graphical abstract. Subsequently, the body mass (g) and body length (mm) of captured individuals were measured and recorded. Afterward, the mouse samples were disinfected with 75% alcohol on the disposable surgical drape and were dissected, then the intestinal contents were collected and stored in sterile freezing tubes containing DNA preservation solution; the contents of the foregut and hindgut were both included. Finally, all samples were stored at −20°C until subsequent metagenomic sequencing. All samples were obtained following the regulations for the implementation of the Wildlife Protection Law of the People’s Republic of China. All our manipulation was approved by the Chengdu Management Branch of Giant Panda National Park.

### Vegetation index collection

The vegetation index (Normalized Difference Vegetation Index, NDVI) data with a 16-day time interval of the study site in 2022 and 2023 were obtained from the Land Processes Distributed Active Archive Center (https://lpdaac.usgs.gov/products/MOD13Q1 v061). The spatial resolution is 250 m. In both 2022 and 2023, 24 16-day time intervals were used. Missing NDVI values were estimated using the Inverse Distance Weighted spatial interpolation method in ArcGIS. Moreover, as the downloaded data are stored values, the actual NDVI was calculated by applying the scale factor (0.0001). The annual average NDVI was then calculated using the Cell Statistics tool in ArcGIS (version 10.8).

### Sample processing for metagenomic sequencing

We excluded juvenile, subadult, and pregnant individuals first according to body mass, body length, and physiological state. A total of 121 samples were selected for sequencing. According to the *Fauna of Sichuan*, the head-body length of adult *Apodemus draco* generally ranges from 82 to 115 mm, so individuals with a head-body length ≥ 80 mm were identified as adults, and individuals with extremely low body weight were excluded (27). DNA extractions were performed using the Tiangen DNA Stool Mini Kit (Tiangen Biotech Co., Ltd., China) according to the manufacturer’s instructions. The concentration and integrity of DNA were measured using the NanoDrop (Thermo Fisher Scientific Inc., Wilmington, DE, USA) and 1% agarose gels. Metagenome library preparation was conducted in one DNA paired-end library with an insert size of 350 base pairs (bp) for each sample following the manufacturer’s instructions (NEB Next Ultra DNA Library Prep Kit, Illumina, NEB, USA). Then, the sample was sent to Novogene (Beijing, China) and sequenced on an Illumina NovaSeq 6000 platform adopting a 150 bp PE sequencing strategy. Our raw reads were filtered to remove low-quality sequences, including those containing (i) adapter contamination, (ii) more than 50% continuous bases whose Phred quality was lower than 5, and (iii) more than 10% (unrecognizable nucleotide) N bases. Then, clean reads are aligned to the host reference genome by using Bowtie2 version 2.4.5 for the host contamination removal (28). Due to the lack of the whole genome data of *A. draco*, we used the reference genome of *A. draco* relatives (*Apodemus sylvaticus*), obtained from the NCBI database (https://ftp.ncbi.nlm.nih.gov/genomes/all/GCF/947/179/515/GCF_947179515.1_mApoSyl1.1/GCF_947179515.1_mApoSyl1.1_genomic.fna.gz).

### Metagenomic taxonomic and functional annotation

We identified and quantified microbiomes in all gut metagenomic samples using Kraken2 version 2.1.3 based on a database that encompasses bacteria, archaea, viruses, fungi, and protists (29). Subsequently, we utilized Bracken version 2.9 to reallocate the identified and quantified results obtained from Kraken2, ensuring that all counts were assigned to species-level microbiomes (30). Finally, the results of all samples were organized by taxonomic levels and merged into a final microbial abundance table.

We performed separate assembly of clean reads for each metagenomic sample using MEGAHIT version 1.2.9 to obtain contigs (31). Gene prediction on contigs for each sample was carried out using Prodigal version 2.6.3 (32). Redundant genes were further removed by CD-HIT version 4.8.1 according to 95% similarity and 90% coverage (33). Finally, nucleotide sequences were translated into amino acid sequences. Subsequently, we used EggNOG-mapper version 2.1.12 to infer orthologous genes related to Archaea and Bacteria in the non-redundant amino acid sequences based on the eggNOG database and obtained the corresponding GO functional descriptions as annotation results (34). The non-redundant amino acid sequences were aligned against the Carbohydrate-Active enZYmes (CAZYmes) database using dbCAN version 4.1.0 (35). Finally, we used Salmon version 1.10.2 to quantify the abundance of non-redundant genes in the metagenomic data set, and gene abundance was normalized using Transcripts Per Million (TPM) (36). Additionally, after removing host sequences, clean reads were used with HUMAnN3 version 3.0.1 for metabolic pathways, resulting in annotation results from the MetaCyc Metabolic Pathway Database (37).

### Statistical analysis

The observed species, Shannon diversity, and Pielou index of the gut microbiome were calculated based on the abundance of species level in each sample by the *Vegan* package within R (version 4.4.0). We modeled each alpha diversity metric using generalized additive models (GAMs) in the R package *mgcv* and piecewise linear regression analysis (PLR) with the R package *segmented* to explore the relationship between alpha diversity and altitude and identify breakpoints. Meanwhile, GAMs and/or PLR were established to examine the relationship between NDVI in quadrat, the phylum-level relative abundance of gut microbiome, the abundance of CAZymes, and altitude. Additionally, in our construction of a GAM for the relationship between the number of samples captured and altitudinal gradient, because the altitudinal gradient is an ordinal categorical variable with equal intervals, we converted the altitudinal gradient into Arabic numerals to facilitate modeling, “1,400–1,500 m” to “above 3,900 m” correspond to “1” to “26.” In all GAMs, we modeled the smooth terms for explanatory variables with cubic regression splines (bs = “cr”). The knots were selected by calculating the *gcv errors* for each candidate model and choosing the knots corresponding to the model with the smallest *gcv errors*. Then, the fitted GAM was diagnosed using *gamcheck* in R (*P* > 0.05). Also, the correlation between altitude and the relative abundance of gut microbiome and microbial functions was based on Spearman Correlation Analysis in the R package *Psych*. All figures were visualized using the R package *ggplot2* and *heatmap*.

Microbial co-occurrence networks under different altitudinal gradients in the gut of *A. draco* were constructed using Spearman correlations (R package: *WGCNA*, function: corAndPvalue) (38). The altitudinal gradients were categorized into six strata: 1,400–1,800 m, 1,800–2,200 m, 2,200–2,600 m, 2,600–3,000 m, 3,000–3,400 m, and above 3,400 m. Robust correlations with *r* > |0.60| and adjusted *P* value < 0.01 (adjusted by the Benjamini–Hochberg procedure) were selected to construct networks (39). Networks were visualized in the Gephi software (version: 0.10.1) (40). The topological characteristics of networks were calculated in the statistics tools in Gephi, including the number of nodes and edges, average degree, modularity, and the percentage of positive/negative correlations. The calculation of network robustness (the proportion of remaining species in the networks after random node removal) followed the methods of Yuan et al. (41). One hundred random networks were generated by randomly removing 50% of the nodes from the original network in each of 100 iterations. Meanwhile, we established cubic polynomial regression models using R (function: poly) to explore the relationship between the robustness of the gut microbiome and altitude. Differences in the robustness of the gut microbiome among different altitudinal gradients were analyzed using Welch’s ANOVA (function: oneway.test) followed by Games-Howell *post hoc* tests (package: PMCMRplus, function: gamesHowellTest) in R4.4.0.

## RESULTS

### Gut microbial factors structuring the altitudinal distribution pattern of *A. draco*

#### Hump-shaped distribution of *A. draco* populations along altitudinal gradients

**Fig 1:**
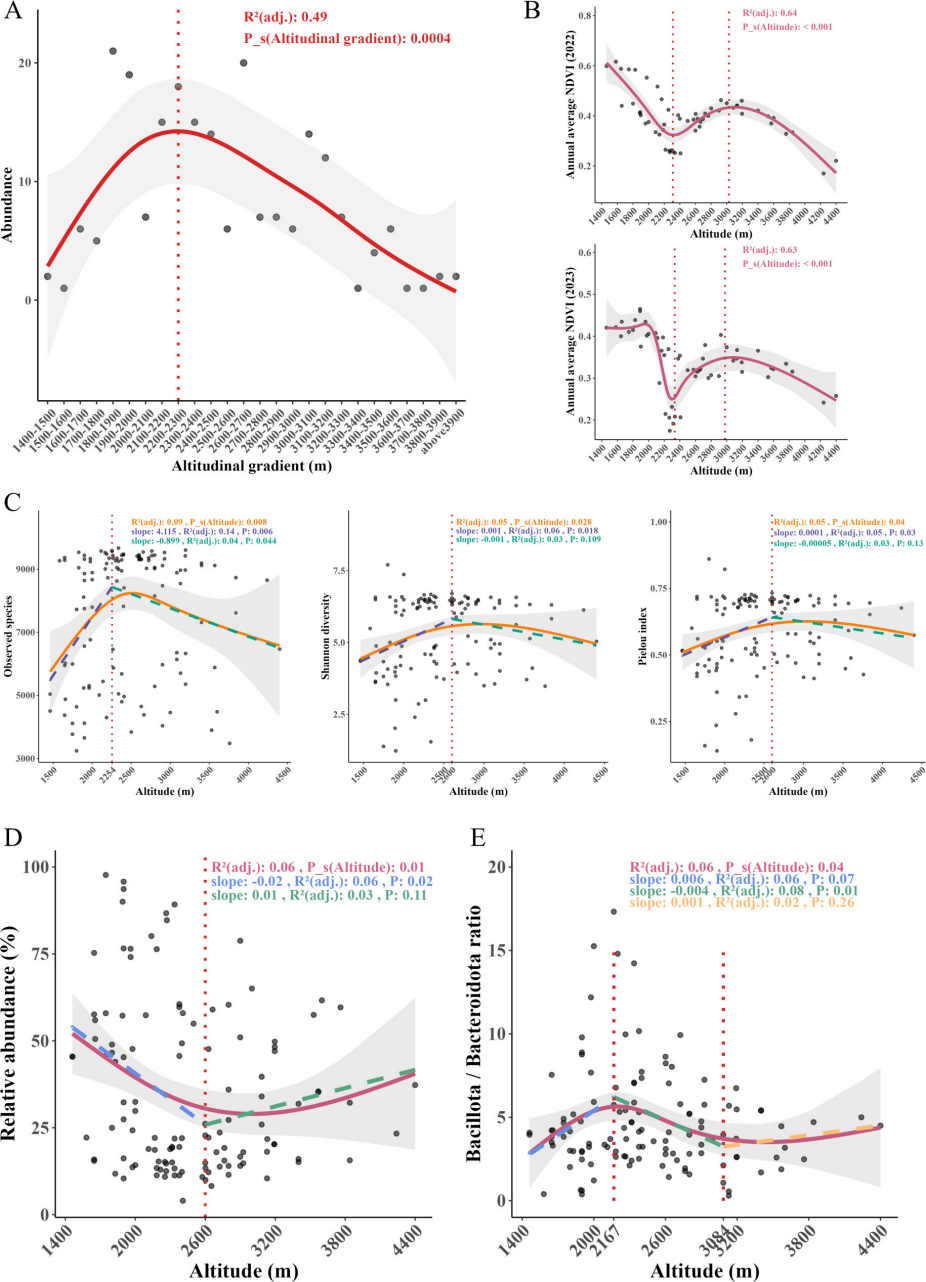
The changes in *A. draco* abundance, NDVI, microbial diversity, and bacterial taxa with altitude. (**A**) Abundance of *A. draco* along the altitudinal gradient. (**B**) Altitudinal distribution pattern of annual average NDVI in 2022 and 2023. (**C**) Altitudinal distribution pattern of microbial alpha diversity in *A. draco* gut. (**D**) Variation in the relative abundance of *Pseudomonadota* across altitudes. (**E**) Altitudinal variation in the Bacillota/Bacteroidota ratio. All nonlinear relationships were fitted using GAM [smooth terms specified by the “s ()” function], and the breakpoints (vertical red dashed line) were estimated using PLR. All linear relationships were fitted using simple linear regression. The letters in different colors correspond to the *R*^2^ and *P* values of the respective color models.

In the study, a total of 219 samples were captured from 1,460 to 4,400 m. We divided the altitudinal gradient into 26 bands, each representing a 100 m altitude interval. The results indicated that the abundance of *A. draco* populations increased and then decreased with the altitudinal gradients (Fig. 1A), with maximum abundance observed at 2,200–2,300 m. Therefore, *A. draco* still displayed a typical hump-shaped distribution along altitudinal gradients. The mid-altitude zone may be the optimal habitat for this species.

#### Changes in vegetation index (NDVI values) at quadrats along altitudes

**Fig 2:**
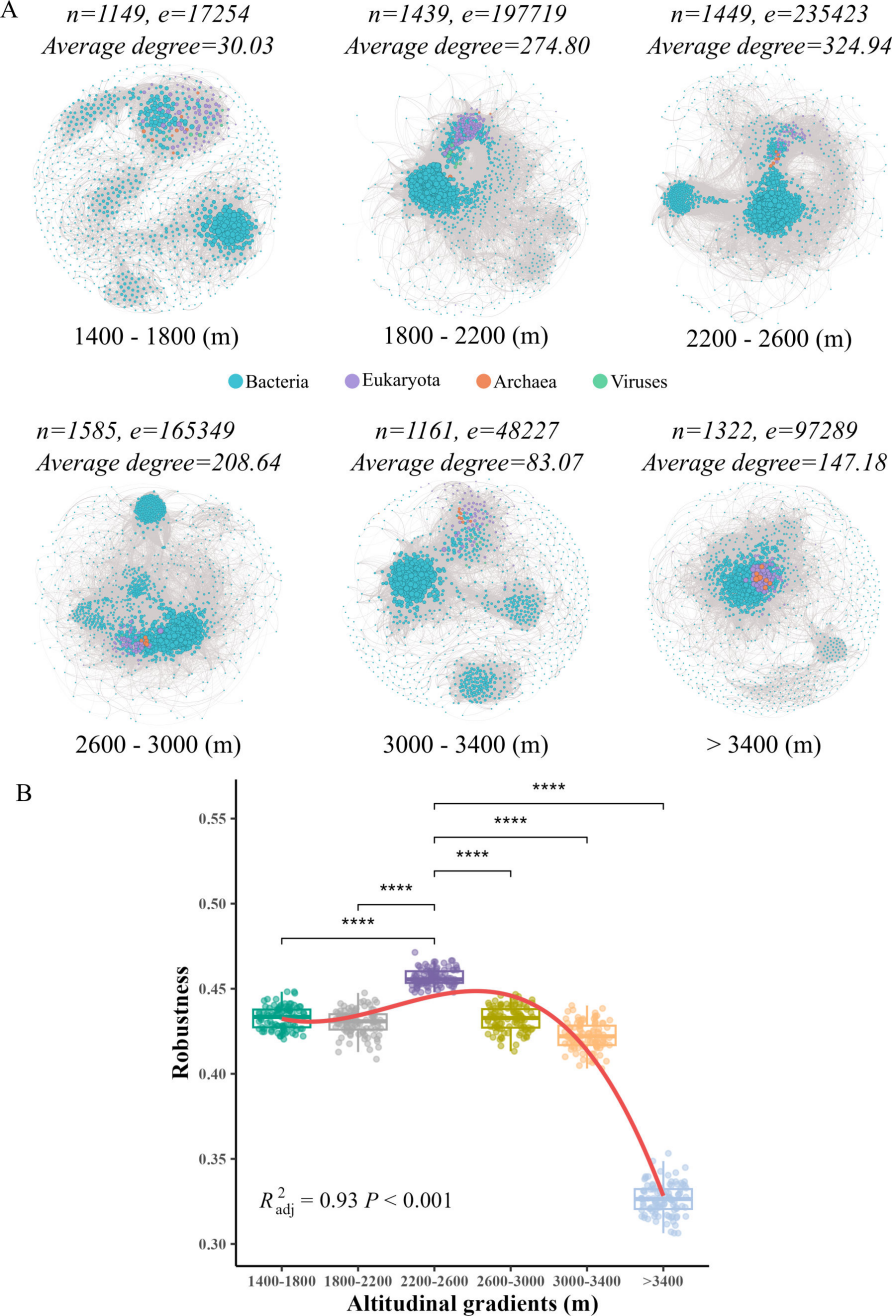
The complexity and stability of gut microbiome along the altitudinal gradient. (**A**) Visualization of microbial network in different altitudinal gradients. Each node represents a microbial species (blue for Bacteria, purple for Eukaryota, orange for Archaea, and green for Viruses), and node size is degree centrality. The thickness of edges is proportional to the correlation coefficient. (**B**) Comparison of network robustness in different altitudinal gradients (measured as the proportion of taxa remaining with 50% of the taxa randomly removed from networks). Nonlinear relationships were fitted using GAM [smooth terms specified by the “s ()” function]. Significant comparisons between 2,200–2,600 m and other altitudinal zones are indicated by ****P < 0.0001.

The annual average NDVI in 2022 and 2023 was calculated for a total of 56 quadrats. The GAM indicated that annual average NDVI in 2022 (Fig. 2B) and 2023 (Fig. 1C) exhibited a three-stage pattern with altitude, characterized by an initial decrease, subsequent increase, and final decrease, and potential breakpoints in NDVI at around 2,300 and 3,000 m, respectively. The results indicated that lower NDVI values correspond to maximum abundance of *A. draco* populations at the 2,300 m altitudinal band.

#### Altitudinal patterns of gut microbial diversity

We analyzed changes in gut microbial α diversity of *A. draco* populations with increasing altitude. The results showed that the observed species, Shannon diversity, and Pielou index of the whole-gut microbiome increased and then decreased with increasing altitude (Fig. 1C), with maximum values in observed species and Shannon diversity at around 2,250 and 2,600 m, respectively. The altitudinal trends of observed species, Shannon diversity, and Pielou index in Bacteria (Fig. S1B), Archaea (Fig. S1E), and Virus (Fig. S1F) were consistent with the whole-gut microbiome. Additionally, the altitudinal trend of observed species in Fungi (Fig. S1D) was also consistent with the results presented herein.

#### Altitudinal variation of *Pseudomonadota* and the *Bacillota***/***Bacteroidota* ratio

Our results indicated that the relative abundance of *Pseudomonadota* decreased and then increased with increasing altitude, and the potential minimum abundance was observed at around 2,600 m (Fig. 1D). Meanwhile, the ratio of the relative abundance of *Bacillota*/*Bacteroidota* (the B/B ratio) exhibited a tripartite altitude-dependent pattern: rising, declining, and rebounding phases. Two potential breakpoints occurred at around 2,200 and 3,000 m, with the maximum value at around 2,200 m (Fig. 1E).

#### Gut microbial co-occurrence network along altitudinal gradients

The gut microbial network analysis showed that the gut microbial co-occurrence networks in *A. draco* exhibited distinct topological properties at different altitudes. By comparing different altitudinal gradients, the mid-altitude zone exhibited a higher number of nodes (1,449), edges (235,423), and average degree (324.94) in the network (2,200–2,600 m) (Fig. 2A). In addition, the nonlinear regression analysis revealed a hump-shaped pattern in network robustness across the altitudinal gradient, and the robustness of the network at mid-altitude gradient (2,200–2,600 m) was significantly higher than both lower and higher altitudinal zones (Fig. 2B).

#### Altitudinal variation in gut microbial CAZymes abundance profiles

**Fig 3:**
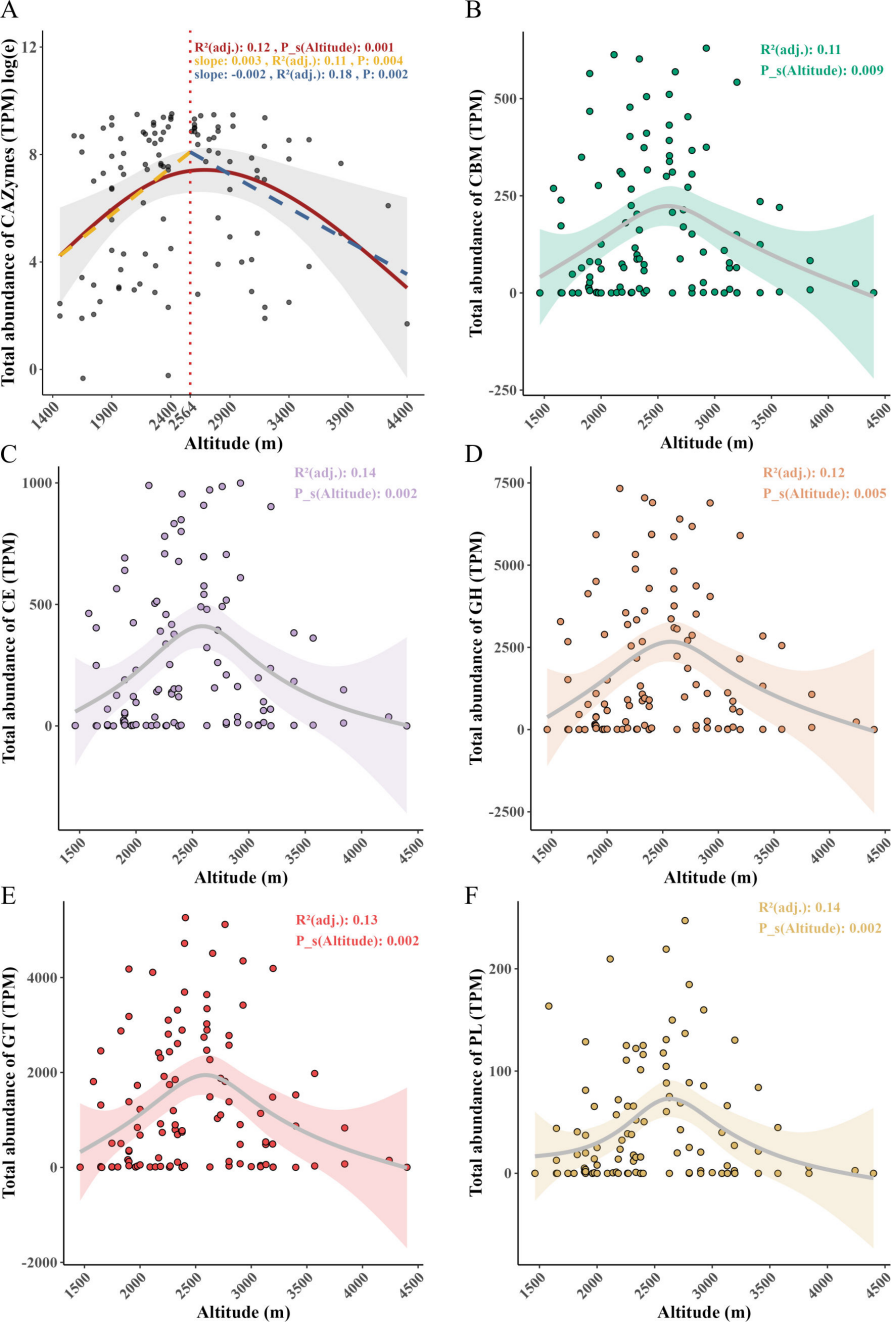
Altitudinal variation of CAZyme abundance. (**A**) Changes in the total abundance of CAZymes/log_e_(TPM) along altitude. (**B–F**) The abundance of five major CAZyme families varied along altitude. All nonlinear relationships were fitted using GAM [smooth terms specified by the “S ()” function], and the breakpoints (vertical red dashed line) were estimated using PLR. All linear relationships were fitted using simple linear regression. The letters in different colors correspond to the *R*^2^ and *P* values of the respective color models.

We annotated six major classes of CAZymes in the gut microbiome of *A. draco*, including auxiliary activity (AA), carbohydrate-binding module (CBM), carbohydrate esterase (CE), glycoside hydrolase (GH), glycosyltransferase (GT), and polysaccharide lyase (PL). The results demonstrated that the total abundance of CAZymes (log_e_TPM) increased first and decreased later with altitude, and the potential peak abundance was at around 2,560 m (Fig. 3A). Meanwhile, the changing trends in the abundances of CBMs, CEs, GHs, GTs, and PLs along altitude were in agreement with the total abundance of CAZymes (Fig. 3B through F). The maximum abundance peak was observed in the mid-altitude zone too.

### Gut microbiota facilitates high-altitude adaptation in *A. draco*

#### Variations in specific gut microbial taxa with increasing altitude

**Fig 4:**
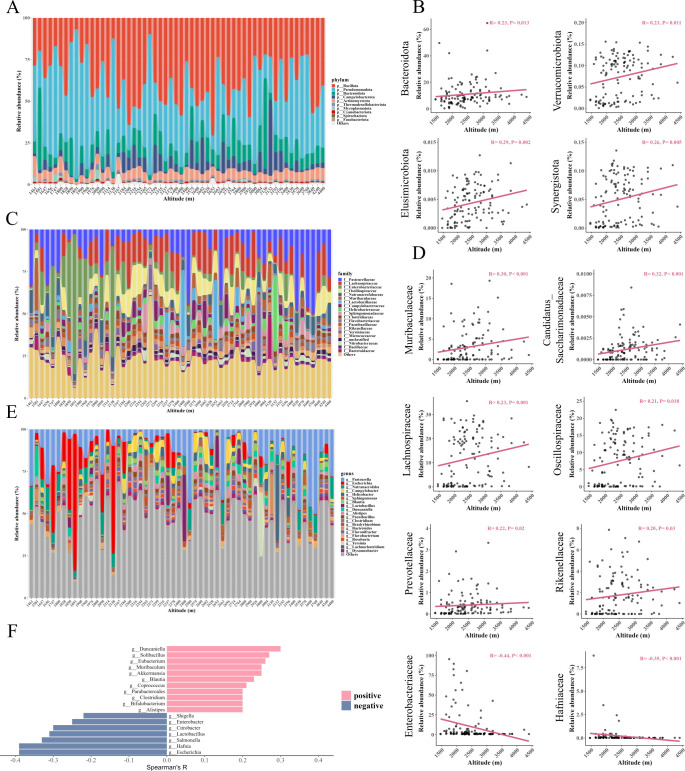
The predominant bacterial taxa at different levels and changes in relative abundance along altitude. (**A**) Predominant taxa at the phylum level. (**B**) Changes at the phylum level in relative abundance along altitude. (**C**) Predominant taxa at the family level. (**D**) Changes at the family level in relative abundance along altitude. (**E**) Predominant taxa at the genus level. (**F**) Changes at the genus level in relative abundance along altitude; pink bar is for positive correlations, and blue bar is for negative correlations. Linear correlations were analyzed using Spearman correlation analysis. Significant difference (*P* < 0.05) is shown here.

We identified altitude-associated microbial taxa through Spearman correlation analysis. In bacteria, the relative abundance increased with increasing altitude in the phyla *Bacteroidota*, *Verrucomicrobiota*, *Elusimicrobiota*, and *Synergistota* (Fig. 4B). Meanwhile, the relative abundance increased with increasing altitude in family *Candidatus_Saccharimonadaceae*, *Rikenellaceae*, *Prevotellaceae*, *Oscillospiraceae*, *Lachnospiraceae*, and *Muribaculaceae*, while *Enterobacteriaceae* and *Hafniaceae* showed negative correlations with altitude (Fig. 4D). At the genus level, the relative abundances of *Duncaniella*, *Solibacillus*, *Eubacterium*, *Akkermansia*, *Muribaculum*, *Blautia*, *Coprococcus*, *Bifidobacterium*, *Parabacteroides*, *Clostridium*, and *Alistipes* showed positive correlations with altitude, while *Escherichia*, *Hafnia*, *Salmonella*, *Citrobacter*, *Shigella*, *Enterobacter*, and *Lactobacillus* showed negative correlations with altitude (Fig. 4F).

The study at the bacterial species level found that relative abundances of the *Bifidobacterium* family (*B. animalis* and *B. adolescentis*) and the *Muribaculaceae* family (*Duncaniella* sp*. C9*, *Duncaniella dubosii*, *Sodaliphilus pleomorphus*, and *Muribaculum gordoncarteri*) showed positive correlations with altitude (Table 1). Meanwhile, the relative abundances of *Dysosmobacter welbionis*, *Akkermansia muciniphila*, *Streptococcus infantis*, *Eubacterium maltosivorans*, *Elusimicrobium minutum*, *Bacillus licheniformis,* and *Blautia wexlerae* also showed positive correlations with altitude (Table 1). Conversely, the results indicated that the relative abundances of the *Escherichia* family (*E. albertii*, *E. coli*, *E. fergusonii*, *E. marmotae*, and *E. albertii*) and the *Shigella* family (*S. flexneri*, *S. dysenteriae*, and *S. boydii*) showed negative correlations with altitude. Meanwhile, the relative abundances of the *Enterobacter* family (*E. huaxiensis*), the *Hafnia* family (*H. alvei* and *H.* sp*. CBA7124*), the *Citrobacter* family (*C. rodentium*, *C. koseri*, *C. freundii*, *C. tructae*, and *C. braakii*), and *Salmonella* family (*S. enterica*) also showed negative correlations with altitude (Table 1).

**TABLE 1:** Changes in gut bacterial taxa at the species level with altitude^*a*^

Correlation	Taxa	Species	*R*	*P* value
Positive correlation	*Bifidobacterium*	*B. adolescentis*	0.25	0.006
		*B. animalis*	0.25	0.005
		*B. bifidum*	0.22	0.015
		*B. pseudolongum*	0.22	0.013
		*B. subtile*	0.31	0.001
	*Muribaculaceae*	*Duncaniella* sp*. C9*	0.25	0.006
		*Duncaniella dubosii*	0.30	0.001
		*Sodaliphilus pleomorphus*	0.25	0.006
		*Muribaculum gordoncarteri*	0.26	0.003
	*Akkermansia*	*A. muciniphila*	0.27	0.002
	*Bacillus*	*B. licheniformis*	0.33	<0.001
	*Eubacterium*	*E. maltosivorans*	0.30	0.001
	*Streptococcus*	*S. infantis*	0.38	<0.001
	*Blautia*	*B. wexlerae*	0.23	0.01
	*Dysosmobacter*	*D. welbionis*	0.24	0.008
	*Eubacterium*	*E. maltosivorans*	0.30	0.001
	*Elusimicrobium*	*E. minutum*	0.26	0.004
Negative correlation	*Escherichia*	*E. marmotae*	−0.44	<0.001
		*E. fergusonii*	−0.39	<0.001
		*E. coli*	−0.37	<0.001
		*E. albertii*	−0.36	<0.001
	*Shigella*	*S. boydii*	−0.25	0.006
		*S. dysenteriae*	−0.33	<0.001
		*S. flexneri*	−0.21	0.022
	*Hafnia*	*H. alvei*	−0.38	<0.001
		*H. paralvei*	−0.21	0.024
		*H.* sp*._CBA7124*	−0.25	0.006
	*Citrobacter*	*C. tructae*	−0.32	<0.001
		*C. rodentium*	−0.28	0.002
		*C. koseri*	−0.30	0.001
		*C. freundii*	−0.27	0.003
		*C. braakii*	−0.27	0.003
	*Enterobacter*	*E. huaxiensis*	−0.27	0.003
	*Salmonella*	*S. enterica*	−0.31	0.001

^*a*^ Spearman correlations between the relative abundance of bacteria at species level and altitude.

Furthermore, the relative abundances of methane-producing archaea (*Methanosarcinaceae*, *Methanomicrobiaceae*, *Methanofollis*, *Methanolobus*, *Methanogenium*, *Methanosalsum*, and *Methanoculleus*) showed positive correlations with altitude (Table S1). However, viruses such as *Poxviridae*, *Herelleviridae*, *Rountreeviridae*, *Betaretrovirus*, and *Andhravirus* showed negative correlations with altitude (Table S1).

#### Altitudinal variations in gut microbial functional profiles

**Fig 5:**
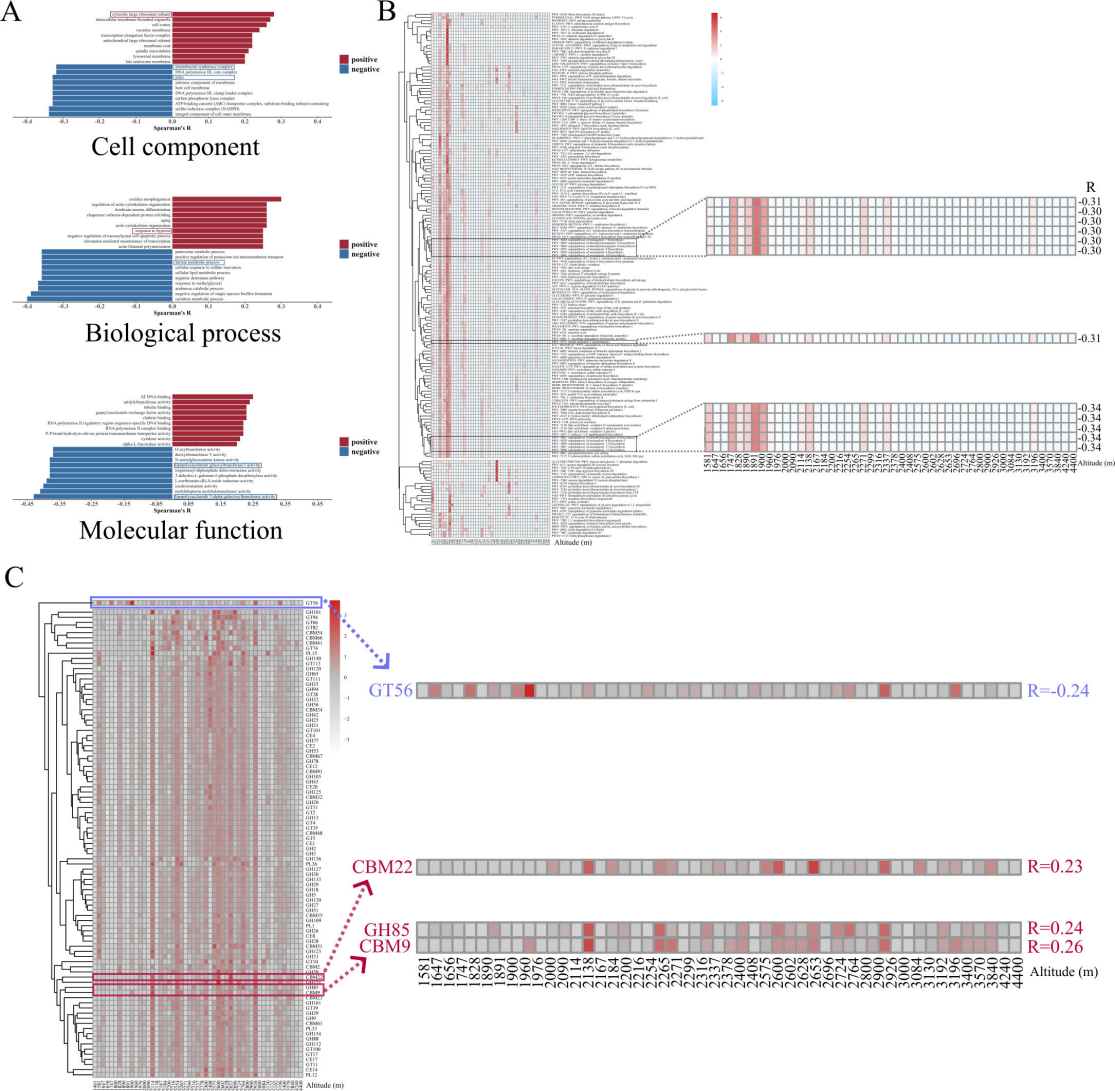
Profiling of gene functions varied along altitude. (**A**) Correlations between the gut microbial GO functions and altitude (top 10 functions with the highest positive/negative correlation); red bar is for positive correlations, and blue bar is for negative correlations. (**B**) Correlations between the gut microbial MetaCyc metabolic pathways and altitude. (**C**) Correlations between the gut microbial CAZyme family and altitude. Correlations were analyzed using Spearman correlation analysis. Significant difference (*P* < 0.05) is shown here.

Our results show that the gut microbiome plays functional roles in host adaptation to high-altitude environments. The relative abundance of cytosolic large ribosomal subunit (GO:0022625) was positively correlated with altitude, while pilus (GO:0009289) and enterobactin synthetase complex (GO:0009366) were negatively correlated with altitude (Fig. 5A). Meanwhile, the relative abundance of response to hypoxia (GO:0001666) was positively correlated with altitude, while lactate metabolic process (GO:0006089) was negatively correlated with altitude (Fig. 5A). Meanwhile, the relative abundances of lipopolysaccharide 3-alpha-galactosyltransferase activity (GO:0008918) and lipopolysaccharide glucosyltransferase I activity (GO:0008919) showed negative correlations with altitude (Fig. 5A).

In metabolic pathways of microbial gene function, the results indicated that the relative abundances of pathways related to menaquinone (vitamin K2) biosynthesis (PWY-5837, PWY-5897, PWY-5898, PWY-5899, PWY-5861, and PWY-5838) and nitrate reduction V (assimilatory) (PWY-5675) showed negative correlations with altitude (Fig. 5B). Additionally, in terms of CAZYmes, the abundance of the family CBM9, GH85, and CBM22 enzymes showed positive correlations with altitude, whereas the family GT56 enzymes were negatively correlated with altitude (Fig. 4E).

## DISCUSSION

Understanding species’ altitudinal distribution patterns and the mechanisms driving these patterns represents a core research topic in ecology (42). For small mammals, the vast majority of research shows that both species richness and diversity exhibit a hump-shaped distribution pattern along the altitudinal gradient (1, 4). Despite extensive research on community-level altitudinal patterns, species-specific distribution patterns are poorly understood. The widespread distribution of *A. draco* across altitudinal gradients offers a unique opportunity to investigate single-species altitudinal distribution patterns. Here, our study reveals that *A. draco* abundance exhibited a distinct hump-shaped distribution pattern along the altitudinal gradient, peaking at mid-altitudes. To further investigate whether gut microbiome participates in the formation of species’ altitudinal distribution pattern through influencing species’ intrinsic adaptive capacity, we conducted a comprehensive analysis of host gut microbiota, evaluating its diversity, stability, composition, and functional profiles.

Diversity is the key to the stability and resilience of gut micro-ecosystems. High diversity means more stable communities and higher levels of functional redundancy, which are essential for the health of the host (43). In the study, the alpha diversity of gut microbiome in *A. draco* exhibits a hump-shaped distribution pattern along altitude that paralleled the species’ abundance distribution, peaking at mid-altitudes. Interestingly, in disagreement with previous studies (greater microbial diversity in high-altitude species [17, 18, 44–46] or in low-altitude species [47]), our study reveals a previously unrecognized pattern. High microbial diversity can improve the fermentation efficiency of diets and provide diverse functions; it would be beneficial for the adaptation of mid-altitude *A. draco* to environments (48, 49).

The gut microbiome is a diverse and complex ecosystem, and microbial stability is vital for host health (43). In the absence of perturbation, the gut microbial community oscillates around a stable ecological state, showing a dynamic equilibrium. Results of the gut microbial network analysis showed that more nodes, edges, and higher average degrees were found in mid-altitude *A. draco* populations. In microbial co-occurrence networks, node count, edge number, and average degree reflect the number of species, interaction complexity, and stability of microbial community, respectively (50, 51), suggesting gut microbial community in *A. draco* at mid-altitude was more complex and stable. Network robustness analysis further revealed that gut microbial robustness also exhibits the mid-peak distribution pattern, and robustness at 2,200–2,600 m was significantly higher than other altitudinal gradients. Additionally, we uncovered a lower abundance of *Pseudomonadota* in the gut of *A. draco* at mid-altitudes, whereas high prevalence of *Pseudomonadota* in the gut is a sign of dysbiosis or an unstable gut microbial community (52). Therefore, the gut microbial community of mid-altitude *A. draco* exhibited higher complexity and robustness than low- and high-altitude populations, indicating superior capacity in the maintenance of healthy homeostasis, as well as stress and perturbation resistance from the external environment.

Our study further suggested that the gut microbiome of the mid-altitude *A. draco* exhibited superior energy harvesting and carbohydrate utilization capacity. In the study, the ratio of the relative abundance of *Bacillota* to *Bacteroidota* exhibited an increasing trend at mid-altitudes. Previous studies in both human and animal models have demonstrated that a higher *Bacillota*/*Bacteroidota* ratio is correlated with enhanced efficiency of energy harvesting from diets (26, 53–55). Therefore, mid-altitude *A. draco* exhibited greater energy harvest efficiency from dietary sources compared to both low- and high-altitude populations. Carbohydrates are the primary source of energy for organisms, and the gut microbiome encodes a vast array of CAZymes, which play a crucial role in the digestion of complex polysaccharides (56, 57). Our study found that the abundance of CAZymes in the gut microbiome exhibited a hump-shaped distribution pattern along altitude, peaking at mid-altitudes (around 2,500 m). This pattern suggests that *A. draco*, which inhabits the mid-altitude habitats, possesses superior abilities to utilize carbohydrates compared to conspecifics at other altitudes. Surprisingly, significantly lower NDVI values were observed at mid-altitudes, coinciding precisely with the maximum abundance of *A. draco*. However, these results directly contradict the positive plant richness or plant productivity-rodent richness correlation (1, 58, 59). NDVI is a metric used to quantify the coverage and health of vegetation. A higher positive value means greater vegetation coverage and vigorous growth of the vegetation. Therefore, higher vegetation coverage tends to provide more habitats and food resources for animals (60). Notably, the *Bacillota*/*Bacteroidota* ratio reaches its maximum under low NDVI conditions. A high ratio could help *A. draco* inhabiting mid-altitude areas with lower vegetation sustain a high-efficiency capacity for energy acquisition from lower food resources, maintaining energy homeostasis, similar to animals inhabiting high-altitude environments (61, 62). Based on the above results, we propose that mid-altitudes may represent an “optimal niche” for *A. draco*, where the interplay between limited resource availability (lower NDVI) and microbial metabolic flexibility creates a trade-off that helps sustain higher species abundance. However, the study is subject to certain limitations. The absolute constraints of environmental factors on species’ distribution and abundance resulted in uneven sample size across altitudinal gradients, with particularly few samples obtained from extremely high altitudes.

Although the altitudinal abundance pattern of *A. draco* peaks at mid-altitudes, individuals of this widespread species are still capable of surviving and reproducing at higher altitudes. High altitude is an extremely resource-scarce environment. Previous data confirmed that *Solibacillus* was enriched in the gut of African buffalo during restricted resource periods. Similar results were also found in goats and cattle with a reduction in forage intake (7, 63, 64). Meanwhile, a higher abundance of *Akkermansia* was observed in fasted Burmese hamsters and the Burmese python, as well as in certain high-altitude species (8, 14, 65, 66). *Akkermansia* and *Akkermansia muciniphila* reside in the gut mucus layer, degrade mucin, and are highly competitive in hosts with low-calorie, nutrient-restricted diets (67). Also, methanogenic archaea (*Methanosarcinaceae* and *Methanomicrobiaceae*) can reduce the accumulation of hydrogen in the gut through the process of methanogenesis and then promote the efficiency of bacterial metabolism (68). In parallel, methane can slow down intestinal motility and reduce chyme transit speed (68). These bacterial taxa are enriched in the gut of high-altitude *A. draco*, potentially contributing to host adaptation under resource-scarce environments. In addition, we identified bacteria involved in lignocellulose degradation, such as *Duncaniella* (e.g., *Duncaniella dubosii*, *Duncaniella* sp*._C9*) and *Muribaculum* (*Muribaculum gordoncarteri*), which were enriched in high-altitude populations (69). Meanwhile, CBM22 and CBM9 enzymes involved in xylan degradation, as well as GH85 enzymes involved in chitin degradation, were also enriched at high altitudes (70–74). The plasticity of the gut microbiome provides *A. draco* with dietary flexibility, enabling adaptation to high-fiber and chitin-rich diets, as well as to limited food availability at high altitudes.

Hypoxia is also a major challenge faced by animals living at high altitudes. A study in human gut microbiota by Su et al. (75) showed that the abundance of genus *Blautia* increased rapidly and continuously with high-altitude hypoxia exposure, and the experiment with laboratory mice proved that *Blautia wexlerae* ameliorated intestinal injury and maladaptation induced by prolonged hypoxia. The increased abundances of these bacterial taxa and the enrichment of hypoxia response-related gene functions at high-altitude *A. draco* may play a crucial role in supporting host adaptation to low-oxygen environments. Moreover, the decrease in the relative abundance of *Lactobacillus* and vitamin K biosynthesis with increasing altitude in the study could be attributed to decreasing oxygen levels (76).

Short-chain fatty acids (SCFAs) derived from gut microbiota are not only an energy source but also play roles in regulating physiological metabolism and immune response and maintaining intestinal barrier integrity (77, 78). Multiple studies consistently indicate that SCFA-producing bacteria were enriched in the gut microbiota of mammals at high altitudes (20, 61, 79, 80). Simultaneously, both hypoxia and cold exposure can result in the enrichment of SCFA-producing bacteria in the intestinal tract (81, 82). In the study, SCFA-producing bacteria, such as *Muribaculaceae*, *Lachnospiraceae*, *Oscillospiraceae*, *Prevotellaceae*, *Rikenellaceae*, *Eubacterium*, *Bifidobacterium*, and *Coprococcus,* were enriched in the gut of high-altitude populations (75, 83, 84), which helps *A. draco* cope with the hypoxic and cold conditions.

Exposure to high-altitude environments also can subject animals to elevated physiological and metabolic stress, which may in turn compromise immune function and increase the risk of infections (85). Opportunistic pathogens can cause disease when the host’s resistance is weakened or due to gut microbial dysbiosis. Interestingly, we found that opportunistic pathogens, such as *Enterobacteriaceae* (86), *Escherichia*, *Salmonella*, *Citrobacter*, *Enterobacter*, and *Shigella,* showed lower relative abundances in high-altitude *A. draco* populations. Han et al. (87) also found a decrease in opportunistic pathogens in high-altitude people, including *Salmonella enterica*, *Escherichia coli*, and *Shigella*. Meanwhile, the decline in viral abundance (e.g., *Poxviridae*, *Herelleviridae*, *Betaretrovirus*, and *Andhravirus*) and the capacity for virulence factor synthesis (e.g., enterobactin, lipopolysaccharide, and enterobacterial common antigen) at high altitudes suggest a reduced pathogenic potential of the gut microbiome (88–90). In addition, the observed decline in nitrate reduction V (assimilatory) at high altitudes implies a reduced metabolic advantage for pathogenic bacteria in the gut anaerobic environment (91, 92). The reduced pathogen infection risk and morbidity at high-altitude *A. draco* populations alleviates the host’s immune system burden, minimizing energy expenditure on inflammation and allowing more energy to be allocated to essential processes such as cold resistance and hypoxia adaptation.

The gut microbiome and hosts have co-evolved for a long time, forming a mutually beneficial symbiotic relationship and adapting convergently to environmental pressures (93). Our work demonstrates the mid-altitude peak distribution pattern in *A. draco* reveals, for the first time, the contribution of gut microbiome to the spatial distribution pattern of species. Additionally, our study broadens the understanding of the role of gut microbiota in promoting host adaptation to high-altitude environments. Moreover, the functional predictions of microbiome require further validation through future controlled experiments. Controlled experiments with microbiota transplantation would further confirm microbial contributions to fitness.

## Data Availability

The raw metagenomic sequencing data reported in this paper have been deposited in the Genome Sequence Archive in the National Genomics Data Center, China National Center for Bioinformation (GSA CRA024847), which are publicly accessible at https://ngdc.cncb.ac.cn/gsa.

## SUPPLEMENTAL MATERIAL

Figure S1 spectrum.02388-25-s0001.tiff Composition of gut microbiome and the correlations between diversity and altitude.

Figure S2 spectrum.02388-25-s0002.tiff Predominant microbial taxa at different levels in the gut.

Figure S3 spectrum.02388-25-s0003.tiff Changes in relative abundance of gut microbiome at phylum level along altitude.

Supplemental material spectrum.02388-25-s0004.docx Tables S1 to S3; legends for Fig. S1 to S3.

Graphical abstract spectrum.02388-25-s0005.tiff Visual depiction of the study.

ASM does not own the copyrights to Supplemental Material that may be linked to, or accessed through, an article. The authors have granted ASM a non-exclusive, world-wide license to publish the Supplemental Material files. Please contact the corresponding author directly for reuse.
