# Supplementary figures and images for: Gut microbiome plasticity explains the altitudinal distribution pattern and adaptability in a small mammal species (Apodemus draco)
Source: Microbiol Spectr. 2025 Nov 20;14(1):e02388-25. doi: 10.1128/spectrum.02388-25 (PMC12772343; doi:10.1128/spectrum.02388-25)

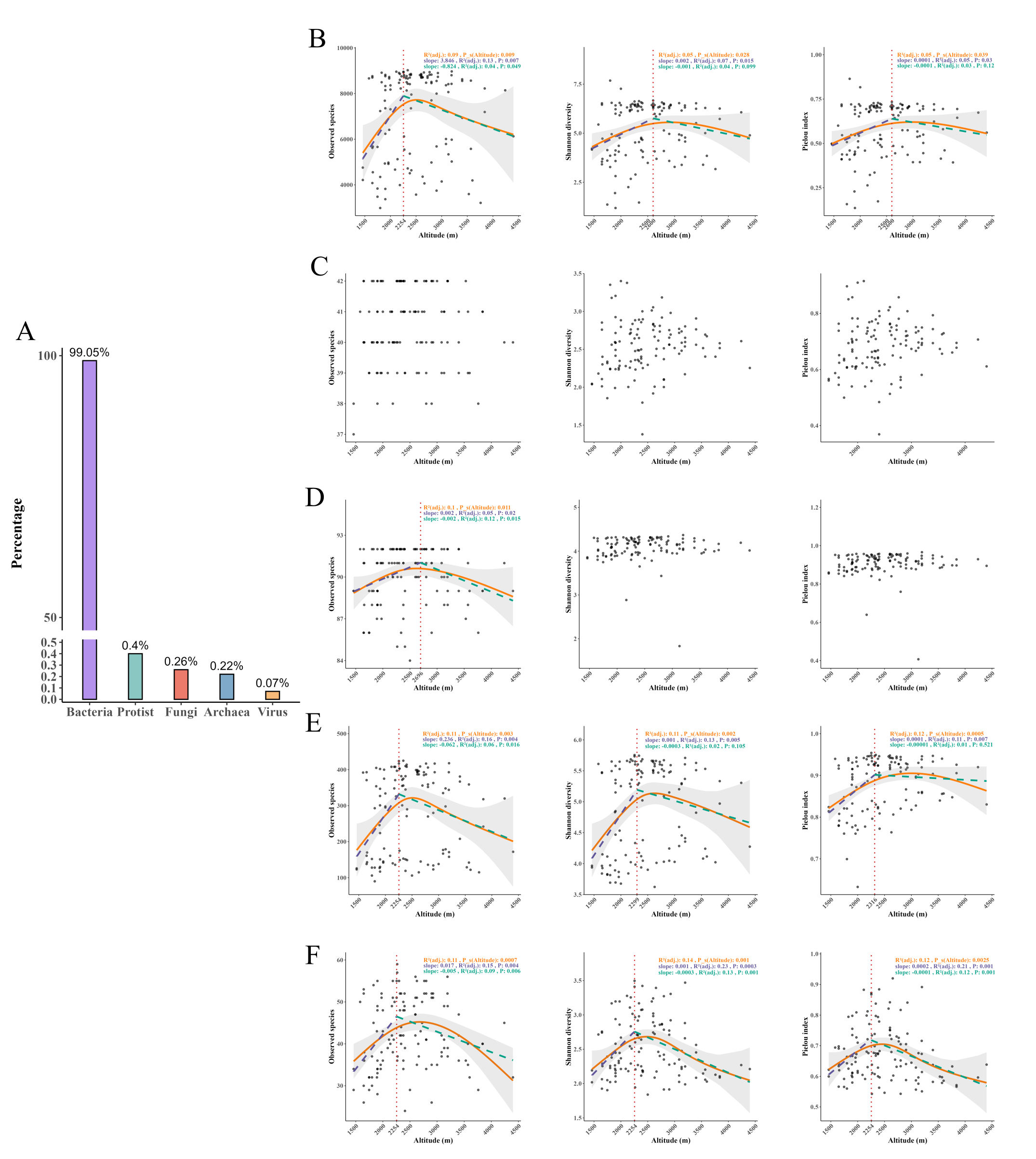

Supplement: Figure S1 — Composition of gut microbiome and the correlations between diversity and altitude. [file spectrum.02388-25-s0001.tiff]

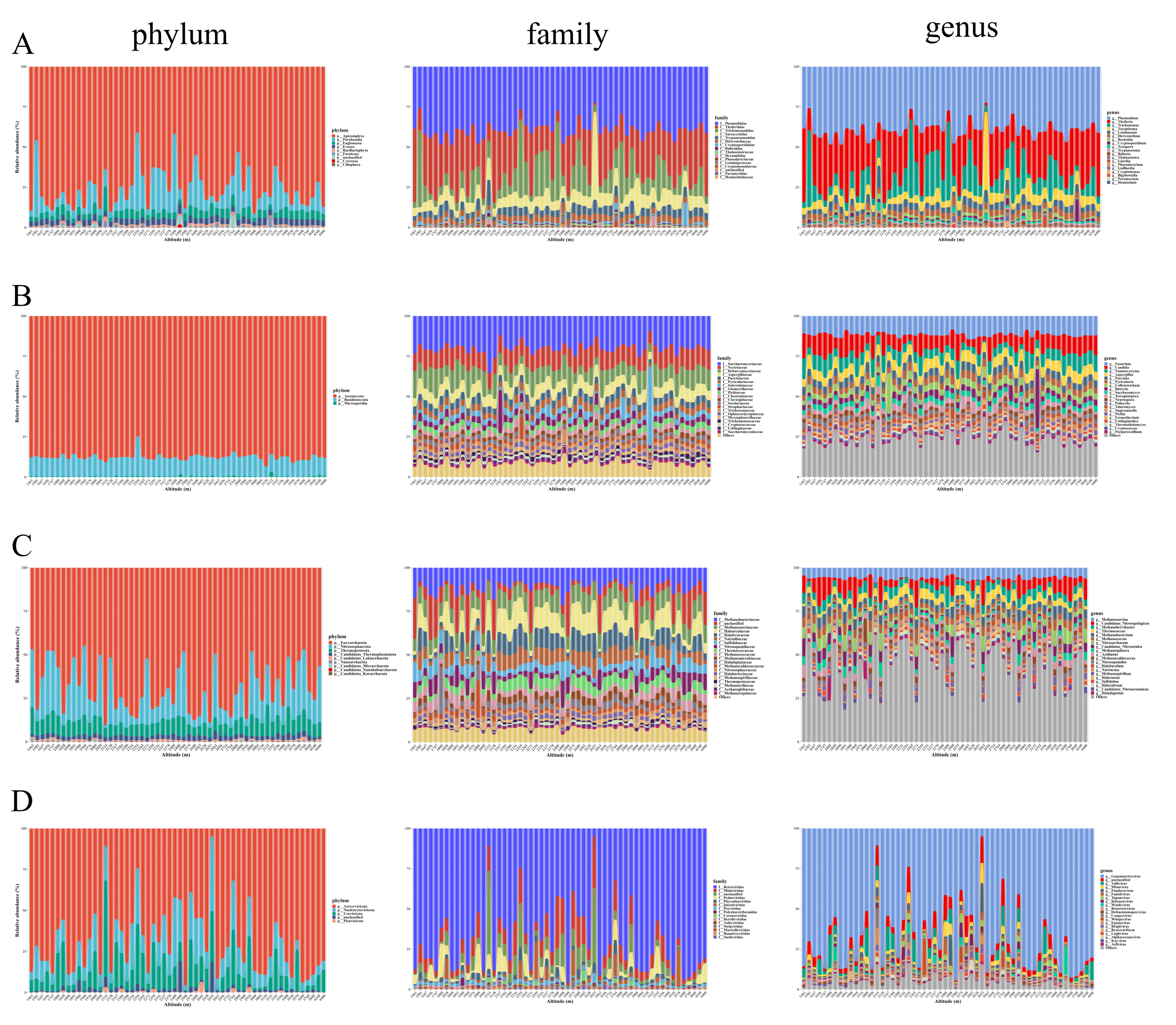

Supplement: Figure S2 — Predominant microbial taxa at different levels in the gut. [file spectrum.02388-25-s0002.tiff]

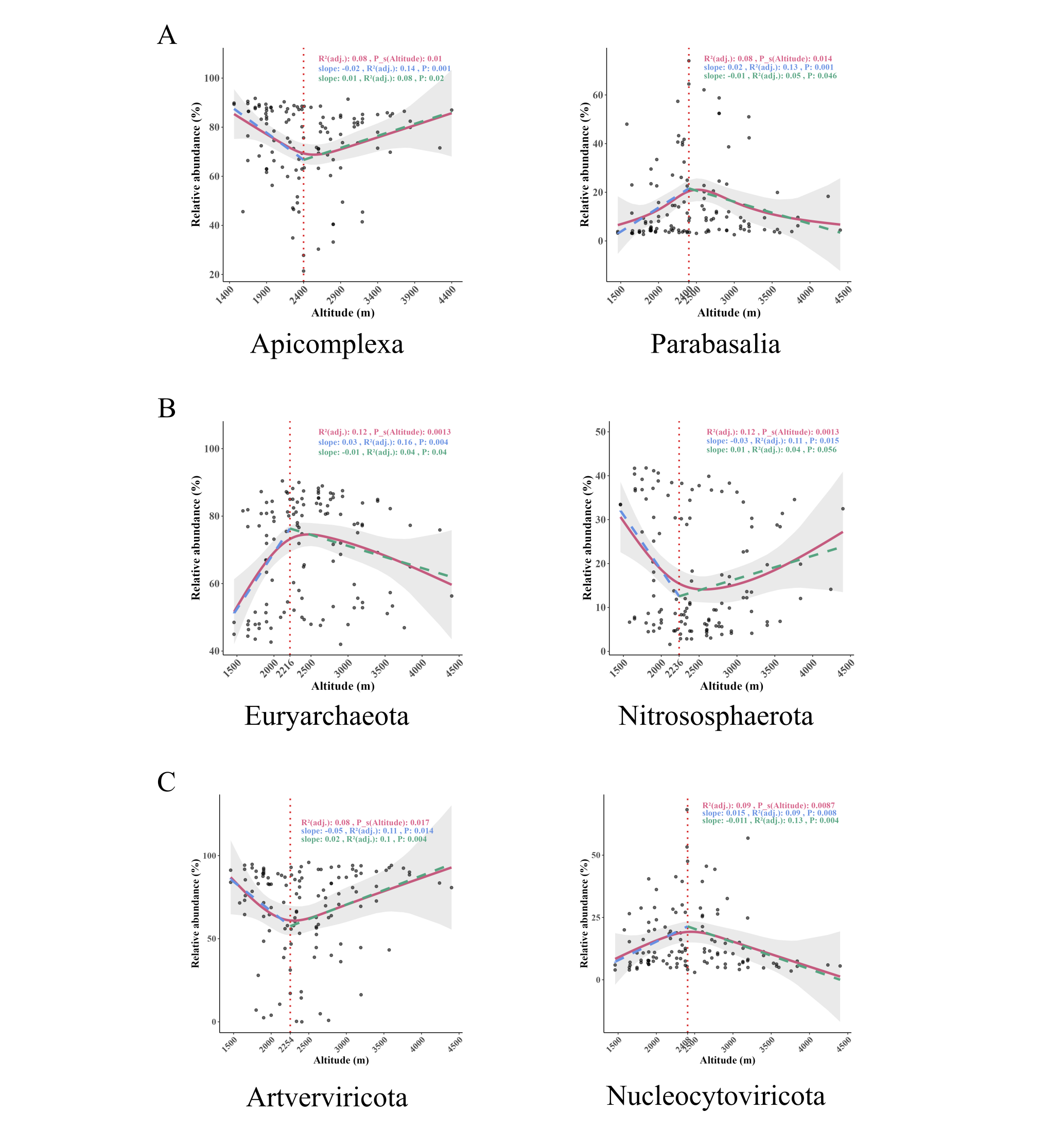

Supplement: Figure S3 — Changes in relative abundance of gut microbiome at phylum level along altitude. [file spectrum.02388-25-s0003.tiff]

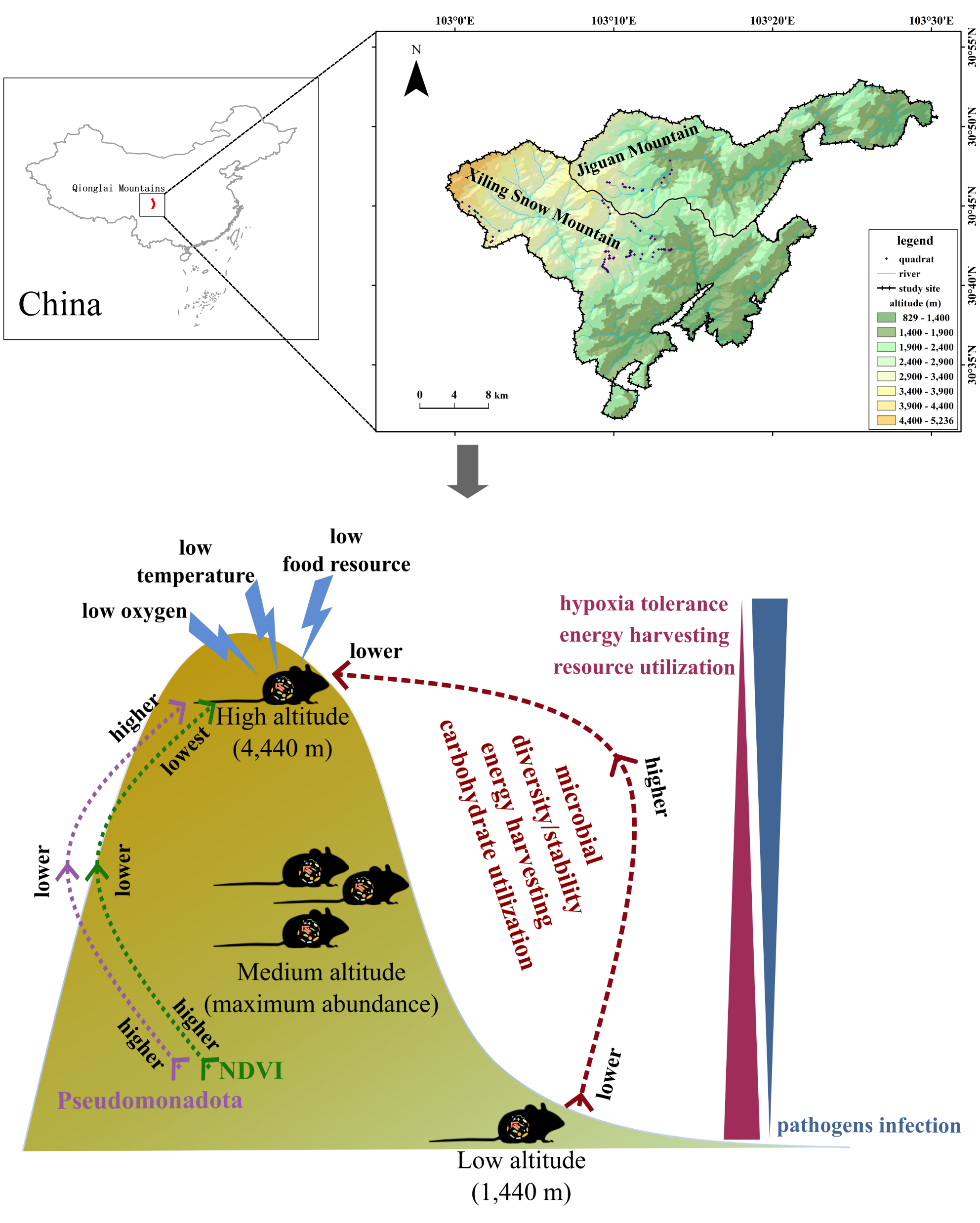

Supplement: Graphical abstract — Visual depiction of the study. [file spectrum.02388-25-s0005.tiff]
