# Supplementary material for: Gut microbiome plasticity explains the altitudinal distribution pattern and adaptability in a small mammal species (Apodemus draco)
Source: Microbiol Spectr. 2025 Nov 20;14(1):e02388-25. doi: 10.1128/spectrum.02388-25 (PMC12772343; doi:10.1128/spectrum.02388-25)
Supplement: Supplemental material — Tables S1 to S3; legends for Fig. S1 to S3. [file spectrum.02388-25-s0004.docx]

**Supplemental Information**

**Gut microbiome plasticity explains altitudinal distribution pattern and adaptability in a small mammal species (*Apodemus draco*)**

Yang Yun^1^, Chao Duan^1^, Xingcheng He^1^, Ruixiang Tang^2^, Yue Lan^2^, Muyang Lu^1^, Tianjiao Liu^1^, Xing Fan^3^, Zhenxin Fan^1, 2, *^, Jianghong Ran^1, *^

^1^ Key Laboratory of Bio-Resources and Eco-Environment of Ministry of Education, College of Life Sciences, Sichuan University, Chengdu 610065, China

^2^ Sichuan Key Laboratory of Conservation Biology on Endangered Wildlife, College of Life Sciences, Sichuan University, Chengdu 610065, China

^3^ Chengdu Management Branch of Giant Panda National Park, Chengdu 610096, China

* Corresponding authors:

**Jianghong Ran**, Key Laboratory of Bio-Resources and Eco-Environment of Ministry of Education, College of Life Sciences, Sichuan University, Chengdu 610065, China. E-mail: [rjhong-01@163.com](mailto:rjhong-01@163.com)

**Zhenxin Fan**, Sichuan Key Laboratory of Conservation Biology on Endangered Wildlife, College of Life Sciences, Sichuan University, Chengdu, 610065, China. E-mail: [zxfan@scu.edu.cn](mailto:zxfan@scu.edu.cn)

**Figure legend**

**Supplementary Figure 1.** The composition of gut microbiome and the correlations between diversity and altitude. (A) Proportions of the major microbial taxa in the gut. Alpha diversity in bacteria (B), protist (C), fungi (D), archaea (F), virus (F) changes with altitude. All nonlinear relationships were fitted using GAM (smooth terms specified by the “s ()” function), and the breakpoints (vertical red dashed line) were estimated using PLR. All linear relationships were fitted using simple linear regression. The letters in different colors correspond to the R² and *P* values of the respective color models.

**Supplementary Figure 2.** The predominant microbial taxa at different levels in the gut. (A) Relative abundance of protistan taxa at the phylum, family and genus level. (B) Relative abundance of fungal taxa at the phylum, family and genus level. (C) Relative abundance of archaeal taxa at the phylum, family and genus level. (D) Relative abundance of viral taxa at the phylum, family and genus level.

**Supplementary Figure 3.** Changes in relative abundance of gut microbiome at phylum level along altitude. (A) Protist. (B) Archaea. (C) Virus. All nonlinear relationships were fitted using GAM (smooth terms specified by the “s ()” function), and the breakpoints (vertical red dashed line) were estimated using PLR. All linear relationships were fitted using simple linear regression. The letters in different colors correspond to the R² and *P* values of the respective color models.

**Supplementary Table 1.** Changes in relative abundance of gut microbial taxa in *A. draco* along altitude

| Taxa | Family | Genus | R | *P* value |
| --- | --- | --- | --- | --- |
| Protist |  | *Neospora* | 0.27 | 0.003 |
|  |  | *Leishmania* | 0.24 | 0.007 |
| Fungi |  | *Psilocybe* | -0.27 | 0.003 |
| Archaea | *Methanosarcinaceae* |  | 0.23 | 0.010 |
|  | *Methanomicrobiaceae* |  | 0.28 | 0.002 |
|  |  | *Methanofollis* | 0.27 | 0.002 |
|  |  | *Methanolobus* | 0.25 | 0.006 |
|  |  | *Methanogenium* | 0.23 | 0.010 |
|  |  | *Methanosalsum* | 0.23 | 0.012 |
|  |  | *Methanoculleus* | 0.22 | 0.014 |
| Virus | *Poxviridae* |  | -0.26 | 0.004 |
|  | *Herelleviridae* |  | -0.20 | 0.028 |
|  | *Rountreeviridae* |  | -0.23 | 0.011 |
|  |  | *Betaretrovirus* | -0.22 | 0.016 |
|  |  | *Andhravirus* | -0.23 | 0.011 |

Spearman correlations between the relative abundance of gut microbial taxa at family / genus level and altitude. Significant difference (P < 0.05) is shown here.

**Supplementary Table 2.** Information on 219 Samples

| Sample_ID | Altitude  (m) | Sex | Body weight  (g) | Head-body length  (mm) | Physiological state (pregnant/nonpregnant/null) |
| --- | --- | --- | --- | --- | --- |
| S0023C | 3216 | female | 18.32 | 83 | nonpregnant |
| S0024C | 2000 | female | 28.56 | 100 | pregnant |
| S0028C | 2000 | male | 31.15 | 100 | null |
| S0030C | 1900 | female | 22.65 | 90 | nonpregnant |
| S0031C | 1900 | male | 28.80 | 95 | null |
| S0032C | 1900 | female | 31.18 | 95 | nonpregnant |
| S0034C | 1800 | female | 32.60 | 90 | pregnant |
| S0036C | 1800 | male | 31.92 | 96 | null |
| S0056C | 1800 | male | 39.28 | 104 | null |
| S0059C | 1800 | male | 10.50 | 79 | null |
| S0061C | 1900 | male | 33.33 | 110 | null |
| S0062C | 1900 | female | 27.68 | 100 | nonpregnant |
| S0063C | 1900 | male | 27.93 | 98 | null |
| S0068C | 2000 | male | 31.19 | 112 | null |
| S0069C | 2000 | female | 33.41 | 105 | pregnant |
| S0070C | 2000 | female | 27.82 | 103 | nonpregnant |
| S0074C | 1800 | male | 34.48 | 110 | null |
| S0076C | 1900 | female | 30.78 | 100 | nonpregnant |
| S0077C | 1900 | male | 31.18 | 115 | null |
| S0082C | 2000 | female | 12.76 | 73 | nonpregnant |
| S0095C | 3000 | female | 22.89 | 80 | nonpregnant |
| S0111C | 2800 | male | 28.37 | 90 | null |
| S0112C | 2900 | female | 28.55 | 85 | nonpregnant |
| S0119C | 2800 | male | 29.31 | 100 | null |
| S0120C | 2800 | female | 35.83 | 90 | pregnant |
| S0121C | 2800 | female | 31.56 | 87 | nonpregnant |
| S0125C | 2800 | male | 25.27 | 80 | null |
| S0126C | 2800 | male | 34.04 | 100 | null |
| S0128C | 2900 | male | 30.14 | 85 | null |
| S0138C | 2800 | male | 31.23 | 95 | null |
| S0139C | 2900 | male | 28.76 | 95 | null |
| S0140C | 2900 | female | 26.66 | 95 | nonpregnant |
| S0173C | 2500 | female | 28.37 | 100 | pregnant |
| S0174C | 2500 | male | 29.15 | 105 | null |
| S0181C | 2400 | female | 27.34 | 90 | nonpregnant |
| S0194C | 2400 | male | 28.24 | 100 | null |
| S0195C | 2400 | male | 27.23 | 100 | null |
| S0200C | 2500 | male | 27.57 | 100 | null |
| S0202C | 2600 | male | 30.27 | 100 | null |
| S0203C | 2600 | female | 31.55 | 100 | nonpregnant |
| S0209C | 2200 | male | 29.39 | 100 | null |
| S0213C | 2400 | male | 30.99 | 110 | null |
| S0214C | 2500 | male | 9.54 | 62 | null |
| S0220C | 2600 | male | 27.2 | 100 | null |
| S0434C | 3216 | female | 31.2 | 110 | pregnant |
| S0439C | 3192 | male | 19.67 | 92 | null |
| S0440C | 3192 | male | 20.06 | 91 | null |
| S0443C | 3196 | female | 20.54 | 92 | nonpregnant |
| S0444C | 3196 | female | 30.35 | 105 | pregnant |
| S0449C | 3196 | male | 17.45 | 80 | null |
| S0453C | 3132 | female | 29.14 | 95 | nonpregnant |
| S0465C | 3196 | female | 18.83 | 91 | nonpregnant |
| S0466C | 2090 | male | 31.68 | 106 | null |
| S0470C | 1909 | female | 31.05 | 102 | pregnant |
| S0471C | 1909 | male | 16.21 | 81 | null |
| S0479C | 3196 | female | 13.57 | 79 | nonpregnant |
| S0480C | 3196 | male | 31.28 | 101 | null |
| S0481C | 3196 | male | 24.65 | 96 | null |
| S0485C | 1960 | male | 30.62 | 108 | null |
| S0486C | 1960 | female | 29.22 | 106 | pregnant |
| S0487C | 1960 | male | 22.17 | 96 | null |
| S0489C | 2138 | male | 21.12 | 97 | null |
| S0494C | 2628 | male | 29.97 | 112 | null |
| S0499C | 2926 | male | 32.90 | 112 | null |
| S0500C | 2628 | male | 31.25 | 108 | null |
| S0508C | 2628 | male | 24.81 | 102 | null |
| S0512C | 2628 | female | 29.90 | 109 | nonpregnant |
| S0515C | 2724 | male | 28.15 | 105 | null |
| S0521C | 2724 | female | 18.64 | 83 | nonpregnant |
| S0526C | 2602 | male | 30.44 | 107 | null |
| S0528C | 2602 | male | 29.49 | 95 | null |
| S0533C | 2184 | male | 28.18 | 112 | null |
| S0534C | 2184 | female | 31.95 | 109 | nonpregnant |
| S0535C | 1891 | female | 21.38 | 87 | nonpregnant |
| S0538C | 2184 | female | 30.03 | 111 | nonpregnant |
| S0539C | 2663 | female | 29.31 | 102 | nonpregnant |
| S0543C | 2628 | male | 10.14 | 75 | null |
| S0553C | 2926 | male | 28.37 | 116 | null |
| S0556C | 2696 | female | 28.49 | 102 | nonpregnant |
| S0557C | 1890 | female | 14.57 | 79 | nonpregnant |
| S0558C | 1890 | female | 14.52 | 83 | nonpregnant |
| S0573C | 1656 | male | 24.72 | 104 | null |
| S0575C | 2216 | female | 30.94 | 98 | pregnant |
| S0578C | 2408 | male | 33.43 | 112 | null |
| S0579C | 2337 | female | 25.36 | 97 | nonpregnant |
| S0580C | 2337 | male | 15.91 | 84 | null |
| S0581C | 2408 | male | 29.56 | 103 | null |
| S0582C | 2408 | female | 34.84 | 97 | nonpregnant |
| S0583C | 2408 | male | 30.34 | 104 | null |
| S0584C | 2337 | male | 30.34 | 111 | null |
| S0585C | 2337 | male | 30.70 | 118 | null |
| S0586C | 2337 | female | 36.09 | 104 | nonpregnant |
| S0587C | 2408 | female | 25.93 | 101 | pregnant |
| S0588C | 2337 | male | 19.98 | 87 | null |
| S0589C | 2408 | male | 20.23 | 92 | null |
| S0593C | 1656 | male | 32.19 | 96 | null |
| S0600C | 2600 | male | 30.01 | 112 | null |
| S0602C | 2600 | male | 28.86 | 112 | null |
| S0603C | 2408 | male | 29.00 | 109 | null |
| S0605C | 2408 | female | 23.70 | 101 | nonpregnant |
| S0606C | 2600 | female | 24.56 | 106 | nonpregnant |
| S0609C | 2265 | female | 26.94 | 103 | nonpregnant |
| S0610C | 2299 | male | 32.79 | 105 | null |
| S0611C | 2265 | female | 28.75 | 100 | nonpregnant |
| S0613C | 2271 | female | 29.43 | 105 | nonpregnant |
| S0614C | 2265 | female | 34.89 | 104 | pregnant |
| S0616C | 2265 | male | 34.81 | 114 | null |
| S0617C | 2316 | female | 19.41 | 90 | nonpregnant |
| S0620C | 2299 | female | 32.92 | 101 | pregnant |
| S0625C | 2316 | male | 35.67 | 116 | null |
| S0626C | 2316 | female | 18.41 | 82 | nonpregnant |
| S0628C | 2216 | male | 32.29 | 111 | null |
| S0776C | 3840 | female | 23.95 | 83 | nonpregnant |
| S0777C | 3840 | female | 24.20 | 95 | nonpregnant |
| S0786C | 4240 | female | 15.19 | 80 | nonpregnant |
| S0804C | 4400 | female | 19.12 | 90 | nonpregnant |
| S0819C | 3400 | female | 15.85 | 75 | nonpregnant |
| S0831C | 3400 | male | 21.35 | 92 | null |
| S0832C | 3400 | male | 20.25 | 88 | null |
| S0834C | 3600 | female | 18.02 | 80 | nonpregnant |
| S0848C | 3570 | female | 15.75 | 78 | nonpregnant |
| S0850C | 3570 | male | 18.31 | 75 | null |
| S0851C | 3570 | male | 17.90 | 91 | null |
| S0852C | 3570 | female | 16.44 | 86 | nonpregnant |
| S0864C | 3530 | male | 25.76 | 86 | null |
| S0866C | 3760 | female | 14.79 | 80 | nonpregnant |
| S0878C | 3400 | male | 18.15 | 82 | null |
| S1260C | 1461 | male | 28.79 | 99 | null |
| S1261C | 1461 | male | 31.90 | 105 | null |
| S1271C | 1647 | male | 22.32 | 98 | null |
| S1272C | 1647 | male | 30.05 | 99 | null |
| S1273C | 1647 | female | 27.58 | 102 | nonpregnant |
| S1274C | 1647 | male | 28.26 | 96 | null |
| S1282C | 1581 | male | 29.28 | 104 | null |
| S1287C | 1747 | female | 25.87 | 98 | nonpregnant |
| S1288C | 1747 | male | 31.74 | 98 | null |
| S1292C | 1747 | male | 11.60 | 66 | null |
| S1296C | 1747 | male | 13.67 | 92 | null |
| S1299C | 1747 | male | 15.66 | 79 | null |
| S1303C | 1828 | male | 32.66 | 99 | null |
| S1306C | 1828 | male | 36.12 | 103 | null |
| S1311C | 1976 | female | 24.13 | 98 | nonpregnant |
| S1312C | 1976 | male | 12.20 | 74 | null |
| S1313C | 1976 | male | 32.19 | 114 | null |
| S1314C | 1976 | male | 30.36 | 113 | null |
| S1317C | 1892 | male | 9.54 | 56 | null |
| S1318C | 1892 | male | 9.70 | 70 | null |
| S1319C | 1892 | male | 10.48 | 66 | null |
| S1411C | 2114 | male | 30.11 | 101 | null |
| S1412C | 2114 | female | 11.97 | 76 | nonpregnant |
| S1416C | 2167 | male | 8.29 | 68 | null |
| S1417C | 2167 | male | 8.39 | 61 | null |
| S1418C | 2167 | female | 31.28 | 94 | pregnant |
| S1419C | 2167 | male | 16.67 | 82 | null |
| S1420C | 2167 | male | 16.43 | 79 | null |
| S1423C | 2167 | male | 18.04 | 86 | null |
| S1424C | 2254 | male | 8.28 | 65 | null |
| S1425C | 2254 | female | 25.51 | 91 | nonpregnant |
| S1426C | 2254 | male | 15.75 | 82 | null |
| S1427C | 2254 | male | 8.41 | 64 | null |
| S1437C | 2378 | male | 15.08 | 80 | null |
| S1438C | 2378 | male | 28.09 | 100 | null |
| S1439C | 2378 | female | 30.01 | 97 | pregnant |
| S1440C | 2378 | male | 27.34 | 101 | null |
| S1445C | 2575 | male | 30.72 | 95 | null |
| S1447C | 2575 | male | 16.70 | 78 | null |
| S1449C | 2653 | male | 28.57 | 95 | null |
| S1451C | 2653 | male | 17.08 | 86 | null |
| S1454C | 2653 | male | 31.63 | 103 | null |
| S1457C | 2653 | male | 31.24 | 102 | null |
| S1460C | 2653 | male | 8.66 | 65 | null |
| S1463C | 2764 | female | 27.51 | 62 | nonpregnant |
| S1464C | 2764 | male | 27.94 | 97 | null |
| S1465C | 2764 | female | 28.20 | 99 | nonpregnant |
| S1469C | 2378 | female | 16.33 | 82 | nonpregnant |
| S1471C | 2378 | male | 28.02 | 102 | null |
| S1472C | 2477 | male | 9.65 | 65 | null |
| S1474C | 2477 | male | 29.81 | 101 | null |
| S1487C | 2764 | male | 30.40 | 102 | null |
| S1488C | 2764 | male | 27.10 | 92 | null |
| S1581C | 3130 | male | 28.66 | 95 | null |
| S1583C | 3130 | male | 21.65 | 90 | null |
| S1602C | 3022 | male | 11.41 | 72 | null |
| S1603C | 3022 | male | 10.22 | 54 | null |
| S1604C | 3022 | female | 29.63 | 105 | pregnant |
| S1608C | 3084 | male | 29.01 | 100 | null |
| S1609C | 3084 | female | 27.21 | 87 | nonpregnant |
| S1611C | 3084 | male | 28.02 | 90 | null |
| S1696C | 3298 | male | 26.30 | 95 | null |
| S1699C | 3504 | male | 24.80 | 92 | null |
| S1700C | 1828 | female | 25.13 | 98 | nonpregnant |
| S1701C | 1828 | male | 31.06 | 103 | null |
| S1702C | 1892 | female | 28.04 | 101 | nonpregnant |
| S1703C | 1892 | male | 17.53 | 92 | null |
| S1704C | 1892 | male | 13.63 | 85 | null |
| S1705C | 1892 | male | 15.86 | 83 | null |
| S1706C | 1892 | male | 10.24 | 71 | null |
| S1707C | 1892 | male | 32.55 | 105 | null |
| S1708C | 1976 | male | 33.70 | 107 | null |
| S1709C | 1976 | male | 13.51 | 81 | null |
| S1710C | 2167 | male | 32.19 | 105 | null |
| S1711C | 2167 | male | 8.13 | 63 | null |
| S1712C | 2167 | female | 26.91 | 93.5 | nonpregnant |
| S1713C | 2254 | male | 17.74 | 90 | null |
| S1714C | 2254 | male | 9.15 | 59 | null |
| S1715C | 2254 | male | 28.36 | 102 | null |
| S1716C | 2254 | male | 26.00 | 96 | null |
| S1717C | 3000 | female | 25.20 | 97 | nonpregnant |
| S1718C | 3000 | male | 29.20 | 99 | null |
| S1719C | 3000 | female | 28.90 | 105 | nonpregnant |
| S1720C | 3000 | male | 27.40 | 101 | null |
| S1721C | 3084 | male | 26.95 | 102 | null |
| S1722C | 3084 | male | 26.64 | 95 | null |
| S1723C | 3084 | male | 17.66 | 78 | null |
| S1724C | 3298 | female | 25.10 | 86 | nonpregnant |
| S1725C | 3298 | male | 28.60 | 90 | null |
| S1726C | 3298 | female | 12.60 | 75 | nonpregnant |
| S1727C | 3298 | male | 27.90 | 95 | null |
| S1728C | 3391 | male | 29.70 | 94 | null |

**Supplementary Table 3.** Information on 121 Sequencing Samples

| Sample_ID | Altitude (m) | Sex | Body weight (g) | Head-body length (mm) | Physiological state (pregnant/nonpregnant/null) | Collection_date | latitude_longitude |
| --- | --- | --- | --- | --- | --- | --- | --- |
| S0028C | 2000 | male | 31.15 | 100 | null | 2022/5/28 | 30.716004 N 103.205098 E |
| S0030C | 1900 | female | 22.65 | 90 | nonpregnant | 2022/5/28 | 30.718168 N 103.206537 E |
| S0031C | 1900 | male | 28.8 | 95 | null | 2022/5/28 | 30.718168 N 103.206537 E |
| S0032C | 1900 | female | 31.18 | 95 | nonpregnant | 2022/5/28 | 30.718168 N 103.206537 E |
| S0036C | 1800 | male | 31.92 | 96 | null | 2022/5/28 | 30.715100 N 103.209675 E |
| S0056C | 1800 | male | 39.28 | 104 | null | 2022/5/29 | 30.715100 N 103.209675 E |
| S0061C | 1900 | male | 33.33 | 110 | null | 2022/5/29 | 30.718168 N 103.206537 E |
| S0062C | 1900 | female | 27.68 | 100 | nonpregnant | 2022/5/29 | 30.718168 N 103.206537 E |
| S0063C | 1900 | male | 27.93 | 98 | null | 2022/5/29 | 30.718168 N 103.206537 E |
| S0068C | 2000 | male | 31.19 | 112 | null | 2022/5/29 | 30.716004 N 103.205098 E |
| S0070C | 2000 | female | 27.82 | 103 | nonpregnant | 2022/5/29 | 30.716004 N 103.205098 E |
| S0074C | 1800 | male | 34.48 | 110 | null | 2022/5/30 | 30.715100 N 103.209675 E |
| S0076C | 1900 | female | 30.78 | 100 | nonpregnant | 2022/5/30 | 30.718168 N 103.206537 E |
| S0077C | 1900 | male | 31.18 | 115 | null | 2022/5/30 | 30.718168 N 103.206537 E |
| S0095C | 3000 | female | 22.89 | 80 | nonpregnant | 2022/6/6 | 30.752769 N 103.156192 E |
| S0111C | 2800 | male | 28.37 | 90 | null | 2022/6/8 | 30.741775 N 103.162564 E |
| S0112C | 2900 | female | 28.55 | 85 | nonpregnant | 2022/6/8 | 30.748464 N 103.160803 E |
| S0119C | 2800 | male | 29.31 | 100 | null | 2022/6/9 | 30.741775 N 103.162564 E |
| S0121C | 2800 | female | 31.56 | 87 | nonpregnant | 2022/6/9 | 30.741775 N 103.162564 E |
| S0126C | 2800 | male | 34.04 | 100 | null | 2022/6/10 | 30.741775 N 103.162564 E |
| S0128C | 2900 | male | 30.14 | 85 | null | 2022/6/10 | 30.748464 N 103.160803 E |
| S0138C | 2800 | male | 31.23 | 95 | null | 2022/6/11 | 30.741775 N 103.162564 E |
| S0139C | 2900 | male | 28.76 | 95 | null | 2022/6/11 | 30.748464 N 103.160803 E |
| S0140C | 2900 | female | 26.66 | 95 | nonpregnant | 2022/6/11 | 30.748464 N 103.160803 E |
| S0174C | 2500 | male | 29.15 | 105 | null | 2022/6/14 | 30.731724 N 103.187649 E |
| S0181C | 2400 | female | 27.34 | 90 | nonpregnant | 2022/6/14 | 30.728878 N 103.189367 E |
| S0194C | 2400 | male | 28.24 | 100 | null | 2022/6/15 | 30.728878 N 103.189367 E |
| S0195C | 2400 | male | 27.23 | 100 | null | 2022/6/15 | 30.728878 N 103.189367 E |
| S0202C | 2600 | male | 30.27 | 100 | null | 2022/6/15 | 30.733312 N 103.185099 E |
| S0203C | 2600 | female | 31.55 | 100 | nonpregnant | 2022/6/15 | 30.733312 N 103.185099 E |
| S0209C | 2200 | male | 29.39 | 100 | null | 2022/6/16 | 30.723934 N 103.194346 E |
| S0220C | 2600 | male | 27.2 | 100 | null | 2022/6/16 | 30.733312 N 103.185099 E |
| S0439C | 3192 | male | 19.67 | 92 | null | 2022/7/15 | 30.702037 N 103.157611 E |
| S0440C | 3192 | male | 20.06 | 91 | null | 2022/7/15 | 30.702037 N 103.157611 E |
| S0443C | 3196 | female | 20.54 | 92 | nonpregnant | 2022/7/15 | 30.694851 N 103.165967 E |
| S0453C | 3132 | female | 29.14 | 95 | nonpregnant | 2022/7/17 | 30.702037 N 103.157611 E |
| S0465C | 3196 | female | 18.83 | 91 | nonpregnant | 2022/7/17 | 30.694851 N 103.165967 E |
| S0466C | 2090 | male | 31.68 | 106 | null | 2022/7/18 | 30.704331 N 103.213614 E |
| S0480C | 3196 | male | 31.28 | 101 | null | 2022/7/18 | 30.694851 N 103.165967 E |
| S0485C | 1960 | male | 30.62 | 108 | null | 2022/7/19 | 30.704441 N 103.216687 E |
| S0487C | 1960 | male | 22.17 | 96 | null | 2022/7/19 | 30.704441 N 103.216687 E |
| S0489C | 2138 | male | 21.12 | 97 | null | 2022/7/19 | 30.694248 N 103.194677 E |
| S0499C | 2926 | male | 32.9 | 112 | null | 2022/7/19 | 30.693902 N 103.157183 E |
| S0500C | 2628 | male | 31.25 | 108 | null | 2022/7/19 | 30.683561 N 103.158582 E |
| S0508C | 2628 | male | 24.81 | 102 | null | 2022/7/20 | 30.683561 N 103.158582 E |
| S0512C | 2628 | female | 29.9 | 109 | nonpregnant | 2022/7/20 | 30.683561 N 103.158582 E |
| S0515C | 2724 | male | 28.15 | 105 | null | 2022/7/20 | 30.686610 N 103.154393 E |
| S0521C | 2724 | female | 18.64 | 83 | nonpregnant | 2022/7/20 | 30.686610 N 103.154393 E |
| S0526C | 2602 | male | 30.44 | 107 | null | 2022/7/20 | 30.682216 N 103.159956 E |
| S0528C | 2602 | male | 29.49 | 95 | null | 2022/7/20 | 30.682216 N 103.159956 E |
| S0533C | 2184 | male | 28.18 | 112 | null | 2022/7/21 | 30.701212 N 103.208299 E |
| S0534C | 2184 | female | 31.95 | 109 | nonpregnant | 2022/7/21 | 30.701212 N 103.208299 E |
| S0535C | 1891 | female | 21.38 | 87 | nonpregnant | 2022/7/21 | 30.704262 N 103.219876 E |
| S0538C | 2184 | female | 30.03 | 111 | nonpregnant | 2022/7/21 | 30.701212 N 103.208299 E |
| S0539C | 2663 | female | 29.31 | 102 | nonpregnant | 2022/7/21 | 30.684573 N 103.157153 E |
| S0553C | 2926 | male | 28.37 | 116 | null | 2022/7/21 | 30.693902 N 103.157183 E |
| S0556C | 2696 | female | 28.49 | 102 | nonpregnant | 2022/7/21 | 30.685716 N 103.155294 E |
| S0558C | 1890 | female | 14.52 | 83 | nonpregnant | 2022/7/24 | 30.704335 N 103.220370 E |
| S0573C | 1656 | male | 24.72 | 104 | null | 2022/7/24 | 30.704423 N 103.227118 E |
| S0578C | 2408 | male | 33.43 | 112 | null | 2022/7/25 | 30.697170 N 103.182769 E |
| S0579C | 2337 | female | 25.36 | 97 | nonpregnant | 2022/7/25 | 30.698345 N 103.184159 E |
| S0582C | 2408 | female | 34.84 | 97 | nonpregnant | 2022/7/25 | 30.697170 N 103.182769 E |
| S0584C | 2337 | male | 30.34 | 111 | null | 2022/7/25 | 30.698345 N 103.184159 E |
| S0585C | 2337 | male | 30.7 | 118 | null | 2022/7/25 | 30.698345 N 103.184159 E |
| S0586C | 2337 | female | 36.09 | 104 | nonpregnant | 2022/7/25 | 30.698345 N 103.184159 E |
| S0593C | 1656 | male | 32.19 | 96 | null | 2022/7/26 | 30.704423 N 103.227118 E |
| S0609C | 2265 | female | 26.94 | 103 | nonpregnant | 2022/7/27 | 30.702446 N 103.185355 E |
| S0610C | 2299 | male | 32.79 | 105 | null | 2022/7/27 | 30.703139 N 103.183868 E |
| S0611C | 2265 | female | 28.75 | 100 | nonpregnant | 2022/7/27 | 30.702446 N 103.185355 E |
| S0613C | 2271 | female | 29.43 | 105 | nonpregnant | 2022/7/27 | 30.701144 N 103.184472 E |
| S0616C | 2265 | male | 34.81 | 114 | null | 2022/7/27 | 30.702446 N 103.185355 E |
| S0617C | 2316 | female | 19.41 | 90 | nonpregnant | 2022/7/27 | 30.700510 N 103.183394 E |
| S0625C | 2316 | male | 35.67 | 116 | null | 2022/7/27 | 30.700510 N 103.183394 E |
| S0626C | 2316 | female | 18.41 | 82 | nonpregnant | 2022/7/27 | 30.700510 N 103.183394 E |
| S0628C | 2216 | male | 32.29 | 111 | null | 2022/7/27 | 30.698849 N 103.188275 E |
| S0776C | 3840 | female | 23.95 | 83 | nonpregnant | 2022/7/30 | 30.744547 N 103.014933 E |
| S0777C | 3840 | female | 24.2 | 95 | nonpregnant | 2022/7/30 | 30.744547 N 103.014933 E |
| S0786C | 4240 | female | 15.19 | 80 | nonpregnant | 2022/7/30 | 30.754040 N 103.007583 E |
| S0804C | 4400 | female | 19.12 | 90 | nonpregnant | 2022/8/4 | 30.747609 N 103.004296 E |
| S0831C | 3400 | male | 21.35 | 92 | null | 2022/8/8 | 30.738450 N 103.027534 E |
| S0832C | 3400 | male | 20.25 | 88 | null | 2022/8/8 | 30.738450 N 103.027534 E |
| S0834C | 3600 | female | 18.02 | 80 | nonpregnant | 2022/8/8 | 30.745088 N 103.020568 E |
| S0851C | 3570 | male | 17.9 | 91 | null | 2022/8/10 | 30.744188 N 103.021287 E |
| S0852C | 3570 | female | 16.44 | 86 | nonpregnant | 2022/8/10 | 30.744188 N 103.021287 E |
| S0864C | 3530 | male | 25.76 | 86 | null | 2022/8/12 | 30.742614 N 103.023872 E |
| S0866C | 3760 | female | 14.79 | 80 | nonpregnant | 2022/8/12 | 30.749461 N 103.014976 E |
| S0878C | 3400 | male | 18.15 | 82 | null | 2022/8/11 | 30.738450 N 103.027534 E |
| S1260C | 1461 | male | 28.79 | 99 | null | 2023/6/11 | 30.797633 N 103.225527 E |
| S1261C | 1461 | male | 31.9 | 105 | null | 2023/6/11 | 30.797633 N 103.225527 E |
| S1271C | 1647 | male | 22.32 | 98 | null | 2023/6/13 | 30.783423 N 103.225517 E |
| S1272C | 1647 | male | 30.05 | 99 | null | 2023/6/13 | 30.783423 N 103.225517 E |
| S1273C | 1647 | female | 27.58 | 102 | nonpregnant | 2023/6/14 | 30.783423 N 103.225517 E |
| S1274C | 1647 | male | 28.26 | 96 | null | 2023/6/14 | 30.783423 N 103.225517 E |
| S1282C | 1581 | male | 29.28 | 104 | null | 2023/6/14 | 30.787623 N 103.227433 E |
| S1287C | 1747 | female | 25.87 | 98 | nonpregnant | 2023/6/15 | 30.779678 N 103.219358 E |
| S1288C | 1747 | male | 31.74 | 98 | null | 2023/6/15 | 30.779678 N 103.219358 E |
| S1296C | 1747 | male | 13.67 | 92 | null | 2023/6/19 | 30.779678 N 103.219358 E |
| S1303C | 1828 | male | 32.66 | 99 | null | 2023/6/17 | 30.774381 N 103.216699 E |
| S1306C | 1828 | male | 36.12 | 103 | null | 2023/6/19 | 30.774381 N 103.216699 E |
| S1311C | 1976 | female | 24.13 | 98 | nonpregnant | 2023/6/17 | 30.716004 N 103.206931 E |
| S1313C | 1976 | male | 32.19 | 114 | null | 2023/6/17 | 30.716004 N 103.206931 E |
| S1314C | 1976 | male | 30.36 | 113 | null | 2023/6/17 | 30.716004 N 103.206931 E |
| S1411C | 2114 | male | 30.11 | 101 | null | 2023/7/6 | 30.766678 N 103.197401 E |
| S1419C | 2167 | male | 16.67 | 82 | null | 2023/7/7 | 30.765720 N 103.195898 E |
| S1423C | 2167 | male | 18.04 | 86 | null | 2023/7/8 | 30.765720 N 103.195898 E |
| S1425C | 2254 | female | 25.51 | 91 | nonpregnant | 2023/7/7 | 30.768574 N 103.187274 E |
| S1426C | 2254 | male | 15.75 | 82 | null | 2023/7/7 | 30.768574 N 103.187274 E |
| S1437C | 2378 | male | 15.08 | 80 | null | 2023/7/7 | 30.769348 N 103.183117 E |
| S1438C | 2378 | male | 28.09 | 100 | null | 2023/7/7 | 30.769348 N 103.183117 E |
| S1440C | 2378 | male | 27.34 | 101 | null | 2023/7/8 | 30.769348 N 103.183117 E |
| S1445C | 2575 | male | 30.72 | 95 | null | 2023/7/10 | 30.769679 N 103.179123 E |
| S1457C | 2653 | male | 31.24 | 102 | null | 2023/7/11 | 30.770074 N 103.177296 E |
| S1464C | 2764 | male | 27.94 | 97 | null | 2023/7/11 | 30.772867 N 103.175971 E |
| S1465C | 2764 | female | 28.2 | 99 | nonpregnant | 2023/7/11 | 30.772867 N 103.175971 E |
| S1469C | 2378 | female | 16.33 | 82 | nonpregnant | 2023/7/9 | 30.769348 N 103.183117 E |
| S1471C | 2378 | male | 28.02 | 102 | null | 2023/7/9 | 30.769348 N 103.183117 E |
| S1581C | 3130 | male | 28.66 | 95 | null | 2023/8/11 | 30.774420 N 103.162216 E |
| S1583C | 3130 | male | 21.65 | 90 | null | 2023/8/21 | 30.774420 N 103.162216 E |
| S1608C | 3084 | male | 29.01 | 100 | null | 2023/8/10 | 30.774791 N 103.159557 E |
| S1609C | 3084 | female | 27.21 | 87 | nonpregnant | 2023/8/10 | 30.774791 N 103.159557 E |
| S1611C | 3084 | male | 28.02 | 90 | null | 2023/8/11 | 30.774791 N 103.159557 E |
